# Alcohol dehydrogenase gene *ADH3* activates glucose alcoholic fermentation in genetically engineered *Dekkera bruxellensis* yeast

**DOI:** 10.1007/s00253-015-7266-x

**Published:** 2016-01-08

**Authors:** Anna Judith Schifferdecker, Juozas Siurkus, Mikael Rørdam Andersen, Dorte Joerck-Ramberg, Zhihao Ling, Nerve Zhou, James E. Blevins, Andriy A. Sibirny, Jure Piškur, Olena P. Ishchuk

**Affiliations:** Department of Biology, Lund University, Sölvegatan 35, Lund, SE-223 62 Sweden; Department of Systems Biology, Technical University of Denmark, DK-2800 Lyngby, Denmark; Consulting statistician, Pinnmöllevägen 48, SE-24755 Dalby, Sweden; Institute of Cell Biology, NAS of Ukraine, Drahomanov Street 14/16, Lviv, 79005 Ukraine; Department of Biotechnology and Microbiology, University of Rzeszow, Zelwerowizca 4, Rzeszow, 35-601 Poland

**Keywords:** *Dekkera bruxellensis*, Auxotrophic mutants, Promoters, *TEF1*, Gene expression, *ADH3*, Glucose fermentation

## Abstract

*Dekkera bruxellensis* is a non-conventional Crabtree-positive yeast with a good ethanol production capability. Compared to *Saccharomyces cerevisiae*, its tolerance to acidic pH and its utilization of alternative carbon sources make it a promising organism for producing biofuel. In this study, we developed an auxotrophic transformation system and an expression vector, which enabled the manipulation of *D. bruxellensis*, thereby improving its fermentative performance. Its gene *ADH3*, coding for alcohol dehydrogenase, was cloned and overexpressed under the control of the strong and constitutive promoter *TEF1*. Our recombinant *D. bruxellensis* strain displayed 1.4 and 1.7 times faster specific glucose consumption rate during aerobic and anaerobic glucose fermentations, respectively; it yielded 1.2 times and 1.5 times more ethanol than did the parental strain under aerobic and anaerobic conditions, respectively. The overexpression of *ADH3* in *D. bruxellensis* also reduced the inhibition of fermentation by anaerobiosis, the “Custer effect”. Thus, the fermentative capacity of *D. bruxellensis* could be further improved by metabolic engineering.

**Electronic supplementary material:**

The online version of this article (doi:10.1007/s00253-015-7266-x) contains supplementary material, which is available to authorized users.

## Introduction

The wine and beer yeast *Dekkera bruxellensis* (anamorph *Brettanomyces bruxellensis*) is the main cause of wine spoilage worldwide, thereby causing immense economic losses (Boulton et al. 1996; Fugelsang 1996; Delfini and Formica 2001; Loureiro and Malfeito-Ferreira 2003; Schifferdecker et al. 2014). Infected wines develop distinctive and unpleasant aromas due to volatile phenols produced by this species, also called “Brett’ taints” (Chatonnet et al. 1995) and normally associated with the smell of barnyard, burnt plastic, wet animal and horse-sweat (Licker et al. 1998). This species is also known for its contribution to the flavour composition of Belgium’s Lambic and Gueuze beers (Dequin et al. 2003; Dufour et al. 2003), specialized red wines (Château Musar and Château de Beaucastel), feta cheese and Kombucha tea (Mayser et al. 1995; Teoh et al. 2004). It is often associated with ethanol production plants, where it can act as an ethanol producer (Passoth et al. 2007) or as a spoiler (de Souza Liberal et al. 2007).

Whole genome sequences of 10 *Dekkera/Brettanomyces bruxellensis* isolates originating from wine, beer or soft drinks were recently reported (Curtin et al. 2012; Piskur et al. 2012; Borneman et al. 2014; Crauwels et al. 2014; Valdes et al. 2014; Crauwels et al. 2015), which is a valuable tool to enhance our understanding of this yeast. The ploidy of the sequenced strains ranges from diploids (CBS 2499, VIB X9085, AWRI 1613, MUCL 49865 and ST05.12/48) to triploids (AWRI 1608, AWRI 1499, CBS 6055 and ST05.12/53); the ploidy of the Chilean wine isolate (LAMAP 2480) is not yet available. Comparative genomics surprisingly placed *D. bruxellensis* as a sister species to the methylotrophic yeast species *Pichia* (*Komatagaella*) *pastoris*, *Ogataea angusta/polymorpha* and *Kuraishia capsulata* (Piskur et al. 2012; Curtin et al. 2012; Curtin and Pretorius 2014); these species are aerobic, Crabtree-negative and poor ethanol producers. Despite its phylogenetic position, *D. bruxellensis* is a good ethanol producer, Crabtree-positive and a facultative anaerobic yeast, and it exhibits a fermentative lifestyle even in the presence of excess glucose and oxygen, traits it shares with baker’s yeast *Saccharomyces cerevisiae. D. bruxellensis* was shown to employ a promoter rewiring that was evolved in parallel to *S. cerevisiae* as one of the molecular mechanisms for the development of the ‘make-accumulate-consume’ life strategy (Rozpedowska et al. 2011). Unlike *S. cerevisiae, D. bruxellensis* is more resistant to acidic pH (Rozpedowska et al. 2011). It also utilizes alternative carbon sources, for example, cellobiose and pentoses such as xylose and l-arabinose (Toivola et al. 1984; Galafassi et al. 2011; Moktaduzzaman et al. 2015); these carbon sources are plentiful and inexpensive in lignocellusic feedstocks. This yeast can also utilize nitrate as a sole nitrogen source due to the presence of the genes of the nitrate assimilation pathway coding for nitrate transporter, nitrite and nitrate reductase and nitrate assimilation transcription factors (Woolfit et al. 2007; Steensels et al. 2015). This enables *D. bruxellensis* to outcompete *S. cerevisiae* in industrial fermentations (de Barros Pita et al. 2011), since *S. cerevisiae* is unable to utilize the abundant nitrate in the major biofuel industry substrate sugarcane juice (de Souza Liberal et al. 2007; Vaughan-Martini and Martini 2011). Like *S. cerevisiae*, *D. bruxellensis* can adapt to fermentation inhibitors in lignocellulose hydrolysates (Blomqvist et al. 2011). These traits make *D. bruxellensis* attractive for biofuels.

Because of their importance for food and biofuel, *D. bruxellensis*’s natural strains have been studied for ethanol production. However, genetically engineered strains have yet to be applied to ethanol production. In this study, we developed auxotrophic strains of *D. bruxellensis* (*ura3* and *ura5*) and we analysed the activity of four *D. bruxellensis* promoters (*ADH3, TEF1, GAL7* and *PHO5*). To investigate the effects of the overexpression of alcohol dehydrogenase gene on the fermentative performance of *D. bruxellensis*, we constructed recombinant strains carrying an expression cassette for the *D. bruxellensis ADH3* gene with the selected promoter *TEF1* and analysed them under both aerobic and anaerobic conditions.

## Material and methods

### Strains and growth conditions

Yeast strains used in this study (Y997 (*ura3*), Y1009 (*ura5*) and Y1010 (*ura5*) listed in Table S1, Supplementary Material) were grown at 25 °C in a complete rich medium YPD (5-g/L yeast extract, 10 g/L peptone, 20 g/L glucose, pH 6.2) or a defined minimal medium (1.7 g/L yeast nitrogen base without amino acids and ammonium sulphate, 5 g/L ammonium sulphate) supplemented with glucose (20 g/L) and uracil (50 mg/L) for uracil auxotrophs. During the characterisation of other putative auxotrophic mutants, any single amino acids were supplemented in the minimal medium, following the standard yeast medium protocols. Yeast transformants were selected on solid minimal medium supplemented with glucose (20 g/L). Yeast transformants were further grown in defined minimal medium (1.7 g/L Yeast Nitrogen Base without amino acids and ammonium sulphate, 5 g/L ammonium sulphate) with the supplementation of either glucose (20 g/L), ethanol (10 g/L) or galactose (20 g/L). For phosphate starvation conditions, cells were grown in defined minimal medium (1.5 g/L yeast nitrogen base without amino acids and without phosphate, 5 g/L ammonium sulphate) supplemented with glucose (20 g/L) and KH_2_PO_4_ (0.005 g/L). This medium was designated as YNB + 10 % phosphate. 

The *Escherichia coli* strain TOP10 (Stratagene, Agilent Technologies, Santa Clara, USA) was used in all experiments that needed a bacterial host. The strain was grown at 37 °C in Luria-Bertani (LB) medium (5 g/L yeast extract, 10 g/L NaCl, 15 g/L peptone, pH 7.4). Transformed *E. coli* cells were selected on LB medium containing 100 mg/L of ampicillin.

### Molecular biology techniques

Plasmid DNA isolations from *E. coli* transformants were carried out using a GeneJET Plasmid Miniprep Kit (Thermo Fisher Scientific, Waltham, USA). All the enzymes that were used for cloning (Phusion DNA polymerase, T4 DNA ligase and restriction enzymes) were purchased from Thermo Scientific (Waltham, USA).

### Generation of auxotrophic mutants

Several *D. bruxellensis* strains (Table S1, Supplementary Material) were mutagenized by UV or ethane methyl sulfonate (EMS), following the standard yeast mutagenesis protocols. For each strain, several thousand colonies were screened for auxotrophy. Putative ur*a*3 and *ura5* strains were selected on defined minimal medium supplemented with both uracil (50 mg/L) and 5′- fluoroorotic acid (FOA 1 g/L). One of the identified *ura3* mutants, *D. bruxellensis* Y997, was used in most of the transformation experiments.

### Plasmid construction

All plasmid constructs generated during this study were confirmed by sequencing. All primers used in plasmid construction are listed in Table S2 (Supplementary Material). The *D. bruxellensis URA3* gene was sub-cloned from the genomic DNA (originating from CBS 2499 strain) and re-sequenced; the obtained sequence was deposited at GenBank with the accession number AY964183 (http://www.ncbi.nlm.nih.gov/nuccore/AY964183). The primer pair OL7 and OL8 amplified a 2.2-kb fragment carrying the *URA3* gene, which was digested with both *Sal*I and *Pst*I and then sub-cloned into the plasmid pUC57, which resulted in P892 (Fig. S1 and Table S3, Supplementary Material).

For the overexpression of *D. bruxellensis ADH3* gene (*DbADH3*), the open reading frame of gene gm1.2868_g (Table S5, Supplementary Material) with 300-bp downstream of the ORF was fused by overlap PCR with the *D. bruxellensis* promoter *TEF1* (*DbTEF1*) using the primers DbTEF1-XbaI-sense, DbTEF1-ADH3-antisense, DbADH3-TEF1-sense and DbADH3-SalI-antisense (Table S2, Supplementary Material). The fragment obtained by overlap PCR was then digested with both *Sal*I and *Xba*I and then cloned into P892, which resulted in plasmid P1227 (Fig. S4, Supplementary Material).

### Transformation system

To transform *D. bruxellensis* yeast, a lithium acetate electrotransformation procedure based on previous protocols by Becker and Guarente (1991) and Boretsky et al. (2007) was developed. An overnight culture was inoculated into 200 mL of YPD and grown overnight at 25 °C. The cells were harvested, washed with water and resuspended in LiAc/TE buffer (0.1 M lithium acetate, 10 mM Tris-HCl, 1 mM EDTA, pH 7.5). After incubation (1 h at 25 °C), the cells were pelleted, washed with 1 M sucrose and diluted 1:1 in 1 M sucrose. Aliquots of 100 μL of this suspension were mixed with 10–20 μg of plasmid DNA and transferred into chilled 2-mm electroporation cuvettes. Electroporation was carried out in a Bio-Rad Gene Pulser II (BioRad Laboratories, Hercules, USA) (resistance 100 Ω, capacitance 50 μF, voltage 2.3 kV) with a Bio-Rad Pulse Controller II (BioRad Laboratories, Hercules, USA) included in the circuit. Directly after the pulse, 900 μL of YPD was added. The cells were then transferred into 1.5-mL tubes and incubated at 25 °C for 1 h. After the incubation, the cells were pelleted, washed once with water and were resuspended in 350 mL of water. Then, 100 μL of suspension was plated out on selective medium and incubated at 25 °C for 5–15 days.

### Stability assay of yeast transformants

In this procedure, cells from single colonies of yeast transformants were inoculated into rich nonselective medium (liquid YPD) and grown for 60 generations at 25 °C. Following growth, the cultures were diluted and plated on YPD plates to obtain single colonies. The dilutions that gave rise to 100–200 colonies were replica-plated on selective minimal medium. The transformants, of which 100 % of the colonies remained prototrophic after this type of cultivation, were assumed stable, selected for further analysis and checked by PCR for the presence of desirable constructs.

### Sequencing and bioinformatic analysis

For the promoter expression studies, the sequences of *D. bruxellensis* genes were identified in the *D. bruxellensis* CBS 2499 strain database JGI (http://genome.jgi.doe.gov/Dekbr2/Dekbr2.home.html) using *S. cerevisiae* protein sequences (http://www.yeastgenome.org) as reference running tblastn. The gene promoter sequences were searched for putative binding sites for transcription factors using a homemade Python script. The *D. bruxellensis ADH3* protein sequence was searched for mitochondrial-targeting motifs by pairwise alignment with *S. cerevisiae ADH3* sequence using EMBOSS ClustalW2 (http://www.ebi.ac.uk/Tools/msa/clustalw2/).

The sequencing of all generated constructs was performed by MWG Biotech (Ebersberg, Germany).

### Alcohol dehydrogenase activity assay in cell-free extracts

For alcohol dehydrogenase activity assay, yeast cells were collected from fermentation experiments from early logarithmic growth phase. Cell-free extracts were prepared using glass beads (Sigma-Aldrich, St. Louis, USA, G8772) as described earlier (Ishchuk et al. 2008). The obtained extracts were used for measuring the enzyme activity spectrophotometrically as described by Postma et al. (1989) with some modifications. The reaction mixture with ethanol as a substrate contained 50 mM potassium phosphate buffer pH 7.5, 0.25 mM NAD^+^ and 100 mM ethanol. The reaction mixture with acetaldehyde as a substrate contained 50 mM potassium phosphate buffer pH 7.5, 0.25 mM NADH and 10 mM acetaldehyde. Adding cell-free extract started the reaction. The protein concentration was determined by the Bradford method (Bradford 1976).

### Gene expression analysis

To study the promoter activity, the transcription levels of the corresponding genes were investigated using *D. bruxellensis* strain Y997 transformed with plasmid P892 (prototroph of Y997). Eight clones of this prototroph transformant were inoculated in 50 mL of YNB supplemented with ethanol (10 g/L), KH_2_PO_4_ (0.005 g/L), galactose (20 g/L) or glucose (20 g/L) and were grown at 25 °C until OD_600nm_ 1–1.5. Cells were harvested (3000×g, 10 min, 4 °C), washed once with diethylpyrocarbonate (DEPC)-treated water and stored at −80 °C for further analysis. RNA was isolated using the Ambion PureLink RNA Mini Kit manual (Life Technologies, Carlsbad, USA). The concentration of the RNA was measured by NanoDrop spectrophotometer (Thermo Fisher Scientific, Waltham, USA). One microgramme of isolated RNA was further used for cDNA synthesis using a Superscript III Reverse Transcriptase kit (Invitrogen, Thermo Fisher Scientific, Waltham, USA) with RNaseOUT Ribonuclease Inhibitor and random primers (Invitrogen, Thermo Fisher Scientific, Waltham, USA). SYBR GreenER qPCR SuperMix (Invitrogen, Thermo Fisher Scientific, Waltham, USA) was used with the complementary (cDNA) as template and the specific primers for the genes *ADH3, GAL7, PHO5* and *TEF1* (Table S2, Supplementary Material). PCRs were performed in duplicates using a RotorGene 2000 cycler real-time PCR machine (Corbett Research, Cambridgeshire, UK) with the conditions specified by Invitrogen. The expression of the analysed promoters in each condition was calculated using REST 2009 software v2.0.13 with RG mode (Pfaffl et al. 2002). The α-tubulin gene (*YML085C*) served as an endogenous control (untreated), and its Ct and amplification data were used to normalize each sample. The influence of the media on the promoter’s expression was additionally analysed using Minitab 17.2.1.0 software (Minitab Inc., State College, USA).

### DNA copy number estimation of *D. bruxellensis ADH3* overexpression strains

To detect the DNA copy number of the *ADH3* gene in strain Y997 carrying plasmid P1227 integrated into the genome, the total DNA was isolated using a standard zymolyase and phenol-chloroform extraction from cultures grown in selective medium (YNB). Thirty nanogramme of purified genomic DNA was used as template in a 20-μL PCR reaction using SYBR GreenER qPCR SuperMix (Invitrogen, Thermo Fisher Scientific, Waltham, USA) in triplicates using a RotorGene 2000 cycler real-time PCR machine (Corbett Research, Cambridgeshire, UK). The relative copy number of *ADH3* gene was calculated using ratios of DNA copy number of the *ADH3* to α-tubulin (*YML085C*) genes in transformants versus the parental strain Y997 (Table S8, Supplementary Material).

### Aerobic and anaerobic glucose fermentation in bioreactors

Aerobic batch cultivations were performed in Multifors (Infors HT, Bottmingen, Switzerland) bioreactors, with a working volume of 1 L using minimal defined media (Verduyn et al. 1992) supplemented with 20 g/L glucose as carbon source and 5 g/L ammonium sulphate as nitrogen source. Dissolved oxygen (monitored using an InPro 6800S sensor from Mettler Toledo, Greifensee, Switzerland) was maintained above 30 % using stirrers in a cascade mode, varying the stirring speed between 200 and 1200 rpm, at 25 °C with airflow set at 1 L/min. The pH was maintained at 5 (±0.5) through automatic addition of 2 M KOH and 1 M H_2_SO_4_ and monitored with a 405-DPAS-SC-K8S/225pH sensor (Mettler Toledo, Greifensee, Switzerland). Samples were taken during exponential growth phase at appropriate intervals and the metabolites analysed by HPLC. The cells were centrifuged (2 min, 16.000×*g*) and filtered through a 0.2-μm membrane filter and analysed using an HPLC 1200 Series System (Agilent Technologies, Santa Clara, USA) to determine the relative concentrations of the residual glucose and metabolites (ethanol, acetic acid and glycerol). The HPLC components and column specifications are reported by Dashko et al. (2015). Product yields, specific glucose consumption rates and metabolites production rates were calculated as reported before (van Hoeck et al. 2000).

Anaerobic experiments were performed with the same bioreactors, instead fitted with Norprene tubings (Cole-Parmer, Vernon Hills, USA) to reduce diffusion of O_2_. Fermentors were flashed with N_2_ (<3 ppm O_2_) at a flow rate of 0.1 L N_2_/min at a constant steering speed of 300 rpm. Synthetic minimal media with 2 % glucose was supplemented with 420-mg/L Tween-80 and 10-mg/L ergosterol. Neither amino acids nor pyrimidines were supplemented.

## Results

### Selection of auxotrophic strains and transformation system

Several *D. bruxellensis* strains were mutagenized by UV or ethane methyl sulfonate (EMS), and thousand colonies of each strain screened for auxotrophic mutants. The frequency of obtained auxotrophs was lower than 0.1 %, only in one third of the tested strains we could obtain a limited number of mutants having one or another growth requirement (Table S1, Supplementary Material). Even in the ‘auxotroph-positive’ strains, like Y879 and Y897, which provided several mutants, the frequency of the obtained auxotrophs was lower than 0.1 %. Among all strains used, only strain Y879 (*D. bruxellensis* CBS 2499) was recently sequenced and is diploid (Piskur et al. 2012; Borneman et al. 2014), the ploidy status of the rest of the strains is unknown. On the other hand, diploid and triploid genomes are common among *D. bruxellensis* isolates (Curtin and Pretorius 2014). Thus, causes for low number of the auxotrophic mutants in our collection could be an increased ploidy of some of the strains or particular gene copy number. Indeed, some of the strains studied (Y865, Y901, Y883 and Y891) carry few copies of *URA3* gene (Fig. S2, Supplementary Material). Only in five strains (Y867, Y869, Y871, Y881, Y897) we could obtain FOA-resistant colonies that also exhibited uracil requirement. One of these mutants, Y997 (Table S4, Supplementary Material), originating from parental strain Y881, was sequenced for the *URA3* locus and shown to harbour two point mutations (one single base pair deletion located at +599, one single basepair substitution located at +600) within the *URA3* open reading frame (Fig. S3, Supplementary Material). The Y997 mutant and other FOA-resistant uracil auxotrophs were used in transformation experiments.

**Fig. 1:**
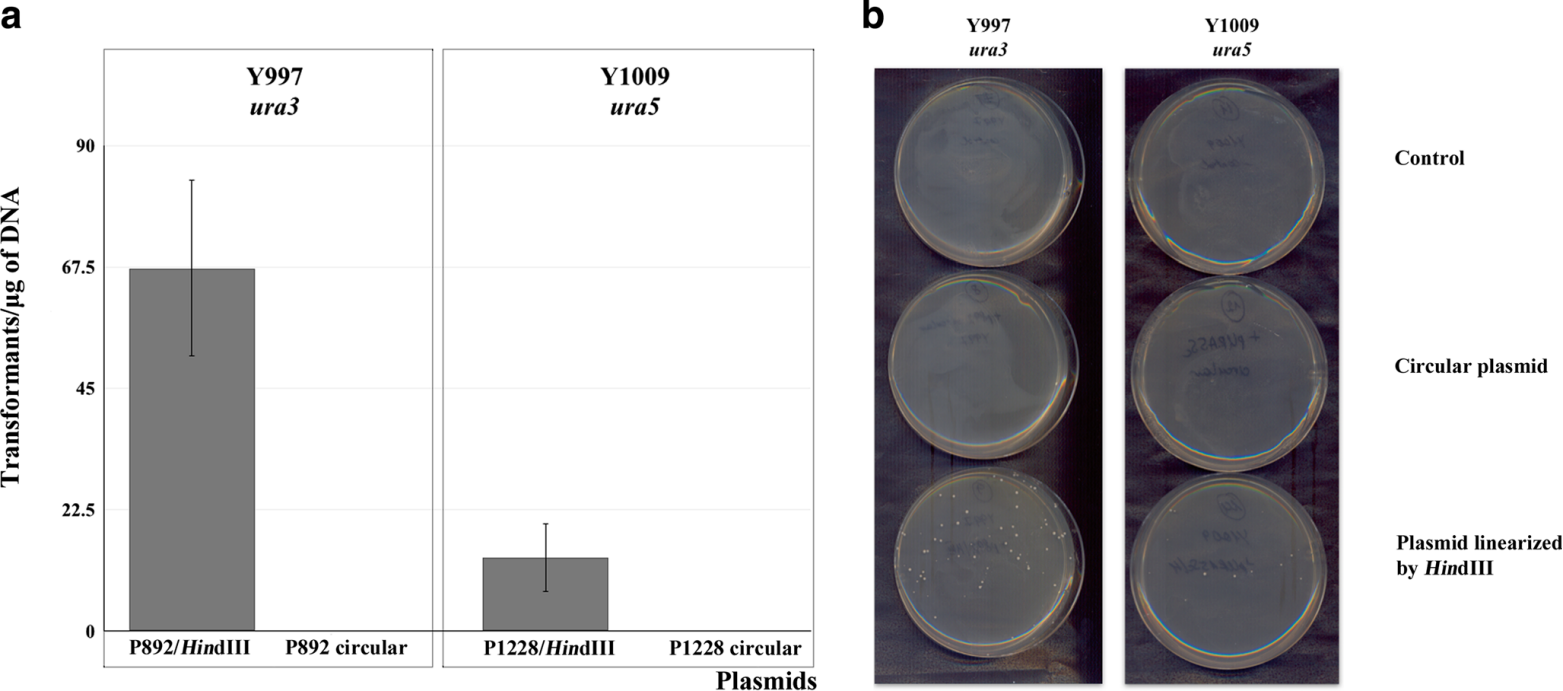
Transformation efficiency of *D. bruxellensis ura3* and *ura5* mutants by linear and circular plasmids. Circular and by *Hin*dIII linearized plasmids were used for transformation. P892 plasmid is carrying *D. bruxellensis URA3* gene, P1228—*S. cerevisiae URA5* gene. **a** Transformation efficiency. **b** Colonies of transformants on selective medium YNB with 2 % glucose

The transformation system for *D. bruxellensis* yeast, which is available, is based on the non-homologous integration using a dominant selective marker (Miklenic et al. 2013). In our study, for transformation procedure of this yeast, we have developed an alternative protocol, lithium acetate-electrotransformation (Materials and Methods), which is based on previous methods described by Becker and Guarente (1991) and Boretsky et al. (2007). This protocol was used to transform auxotrophic mutants obtained in our study. The Y997 *ura3* mutant was transformed with plasmid P892 carrying *D. bruxellensis URA3* gene (Fig. S1, Supplementary Material) linearized with restriction enzyme *Hin*dIII, which is not present within the *URA3* gene. While the linearized P892 plasmid yielded 68 transformants on average between transformation experiments, circular P892 did not give rise to any transformants (Fig. 1). Among 20 randomly picked P892 transformants, 10 transformants proved to be stable after propagation for 60 generations in non-selective medium (YPD) in a transformants stability assay (see Materials and Methods section).

Other selected FOA-resistant mutants required uracil for growth, but did not give rise to any transformants when being transformed with a plasmid carrying *D. bruxellensis URA3* gene. In *S. cerevisiae*, there are two genes (*URA5* and *URA10*) coding for major and minor isozymes of orotate phosphorybosyltransferase, and *URA10* partially complements the mutations in *URA5* gene (de Montigny et al. 1990); thus, 5-FOA-resistant mutants in this yeast are mostly *ura3*. The genome of *D. bruxellensis* CBS 2499 (Y879, http://genome.jgi.doe.gov/Dekbr2/Dekbr2.home.html) was found to carry *URA5*, but lacked *URA10*. Two of the FOA-resistant *D. bruxellensis* mutants obtained in our study from Y871 (CBS 1942) strain (Table S4, Supplementary Material) were proved to be *ura5* by complementation with the *S. cerevisiae URA5* gene with a transformation efficiency of 14 transformants per microgram of DNA (Fig. 1).

### Promoter analysis

To develop an expression plasmid for *D. bruxellensis*, we have experimented with four promoters, which are known to be strong or regulative in other yeast species: *ADH3* (alcohol dehydrogenase isoform III), *TEF1* (translational elongation factor 1A), *PHO5* (acid phosphatase) and *GAL7* (α-d-galactose-1-phosphate uridyltransferase). To evaluate the strength of these promoters, we studied the expression of the corresponding genes using the *D. bruxellensis* strain Y997. One of the three *S. cerevisiae ADH3* orthologs was selected (gm1.2868_g (JGI v2.0), and we further refer to it as *ADH3* (Table S5, Supplementary Material).

To study the effects of carbon sources on the promoter activity, their sequences (1000 bp upstream of ATG) were searched for motifs known to be involved in the response to specific carbon source (Mig1, Cph1, Gal4, Adr1, Cat8) or to phosphate limitation (Pho4) (Thukral et al. 1991; Cheng et al. 1994; Lundin et al. 1994; Treitel and Carlson 1995; Gancedo 1998; Shao et al. 1998; Traven et al., 2006; Weinhandl et al. 2014). The Python script highlighted the occurrence of the Mig1 binding site inside the *ADH3* promoter sequence at position 71 bp. The *GAL7* promoter carries two binding sites for Mig1 at positions 144 and 437 bp as described before (Moktaduzzaman et al. 2015). A putative binding site for the Pho4 transcription factor was found at position 835 bp in the *PHO5* promoter sequence. A binding motif for Gcr1 transcription factor was found at positions 638 and 929 bp in the *TEF1* promoter sequence (Table S9, Supplementary Material).

**Fig. 2:**
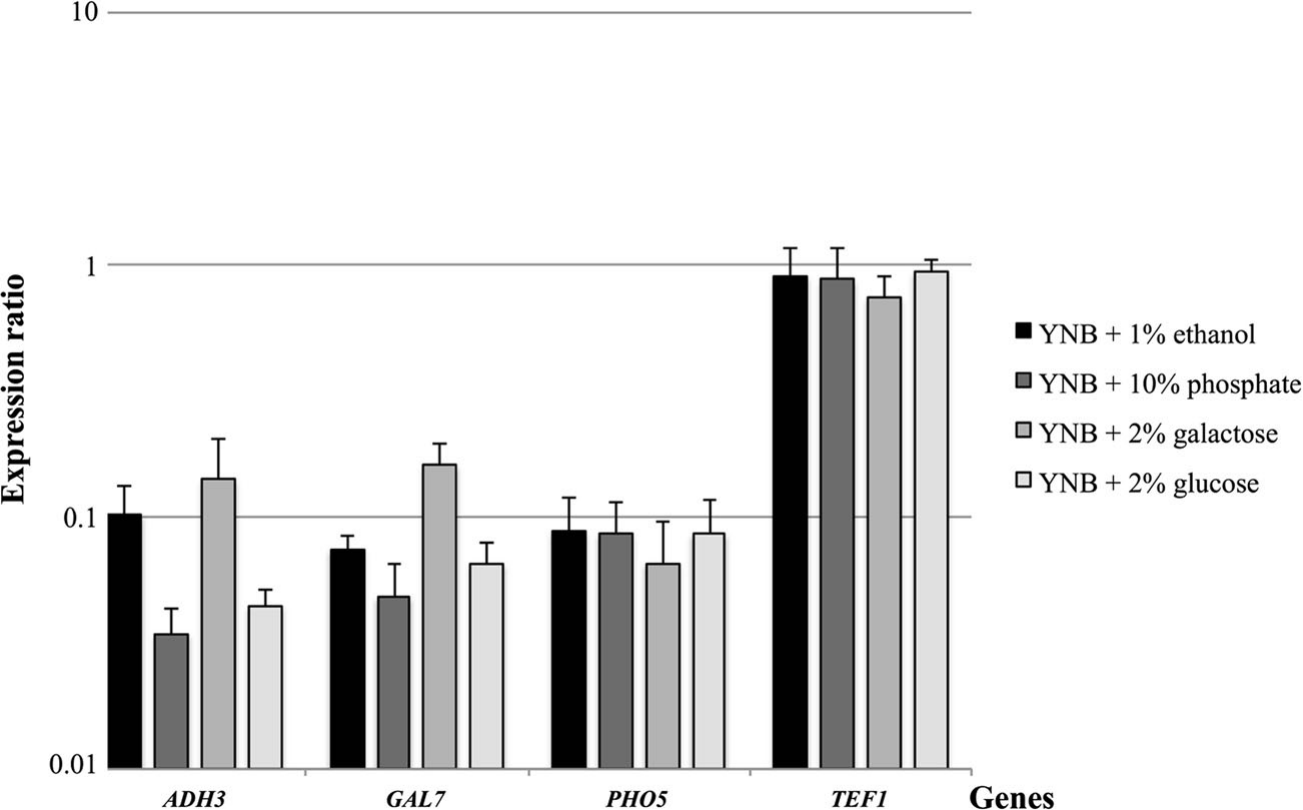
The expression levels of *D. bruxellensis* promoters *DbADH3*, *DbGAL7*, *DbPHO5* and *DbTEF1* in Y997 strain grown in YNB media supplemented with either 1 % ethanol, 2 % galactose, 2 % glucose or phosphate depletion medium (YNB + 10 % phosphate). The *values* shown represent means of the four expression ratios obtained with REST 2009 v2.0.13 software with RG mode from eight clones. The α-tubulin gene Ct and amplification data was used to normalize samples. The *error bars* represent standard deviation. The Y-axis has a logarithmic scale

The selected promoters were studied by analysing the expression of the corresponding genes under conditions of different media by RT-qPCR (Fig. 2, Table S7, Supplementary Material) using primer pairs DbADH3-RT-sense and DbADH3-RT-antisense for *ADH3* gene, DbGAL7-RT-sense and DbGAL7-RT-antisense for *GAL7* gene, DbPHO5-RT-sense and DbPHO5-RT-antisense for *PHO5* gene, DbTEF1-RT-sense and DbTEF1-RT-antisense for *TEF1* gene, α-tubulin-sense and α-tubulin-antisense for *YML085C* gene (Table S2, Supplementary Material). The RT-qPCR reactions of eight prototroph transformants of the Y997 strain were run in duplicates. The study’s dataset was analysed by two approaches (multivariate analysis (Johnson and Wichern 2002) and relative gene expression analysis (Pfaffl et al. 2002)) to estimate the effects of different media on gene expression.

For each of the eight clones, the four media were (deterministically) applied to clone samples, and so the dataset was analysed with a two-way linear model without interaction (Anderson 2003). Clones were modelled as blocks, and media were modelled as treatments. Using this model, the dataset was analysed with MINITAB 17.2.1.0 (Minitab Inc., State College PA, USA). The media had a significant effect on the combined (Ct and Amplification factor) response, according to the trace test of the analysis of variance (ANOVA) (Lawley 1938; Hotelling 1951; Anderson 2003). The media had a significant effect on the gene expression measured by Ct (*p ≤* 0.0005) but not amplification factor (*p* ≈ 0.282), according to the lambda test (Wilks 1932; Anderson 2003). Then, for each gene’s Ct, a univariate ANOVA (without interaction) is reported in Table S6, Supplementary Material. The media factor was insignificant for the genes *YML085C* (*p* ≈ 0.627), *PHO5* (*p* ≈ 0.210) and *TEF1* (*p* ≈ 0.147) and significant for *ADH3* (*p ≤* 0.0005) and *GAL7* (*p ≤* 0.0005) genes. For the genes with significant effects (*ADH3* and *GAL7*), estimates and *t* tests of the effects of the media are shown in Table 1. For the *ADH3* gene, the ethanol and galactose media were highly significant (each *p ≤* 0.0005) in comparison to glucose, which has zero effect by parametrization. For the *GAL7* gene, the galactose medium was highly significant (*p* ≤ 0.0005). The largest effects occurred for the *ADH3* gene Ct, and for this gene, the galactose and ethanol media had large and significant effects (each *p* ≤ 0.0005). For the *GAL7* Ct, the galactose medium had a large and significant effect (*p* ≤ 0.0005) (Table 1). The statistical results provide strong evidence that *ADH3* and *GAL7* are differentially expressed when the medium varies. In contrast, the expression of *YML085C*, *PHO5* and *TEF1* is practically constant regardless of the media (Table 1, Table S6, Supplementary material).

**Table 1:** The effect of particular media on gene expression (Ct) estimated by least squares. No *t* statistics or *p* values are shown for the genes for which media was insignificant, by the preceding analysis of variance

Gene	Medium	Effect	Standard error	*t* statistics	*p* value*
*ADH3*	Constant	22.075	0.469		
	1 % Ethanol	−1.650	0.399	−4.13	0.000
	10 % Phosphate	0.544	0.399	1.36	0.179
	2 % Galactose	−1.843	0.418	−4.41	0.000
	2 % Glucose^a^	0			
*GAL7*	Constant	21.665	0.430		
	1 % Ethanol	−0.587	0.366	−1.61	0.114
	10 % Phosphate	0.563	0.366	1.54	0.130
	2 % Galactose	−1.485	0.383	−3.88	0.000
	2 % Glucose^a^	0			
*YML085C* (α-tubulin)	Constant	16.709	0.491		
	1 % Ethanol	−0.231	0.418		
	10 % Phosphate	−0.088	0.418		
	2 % Galactose	0.332	0.437		
	2 % Glucose^a^	0			
*PHO5*	Constant	21.732	0.568		
	1 % Ethanol	−0.331	0.483		
	10 % Phosphate	−0.075	0.483		
	2 % Galactose	0.726	0.506		
	2 % Glucose^a^	0			
*TEF1*	Constant	16.131	0.38		
	1 % Ethanol	−0.088	0.325		
	10 % Phosphate	0.156	0.325		
	2 % Galactose	0.659	0.340		
	2 % Glucose^a^	0			

*Minitab’s *p*-value “0.000” means that “*p* ≤ 0.0005”

^a^The parametrization forces the YNB medium with 2 % glucose (2 % Glucose) to have zero effect

In the second approach, the REST 2009 software v2.0.13 was used to calculate the gene expression ratios, where *YML085C* was used to normalize the samples. For this program, the blocking (as discussed for ANOVA) was not taken into account, and the ratios of all eight clones obtained were averaged (Fig. 2). It indicates that *ADH3* and *GAL7* are differentially expressed but *PHO5* and *TEF1* appear unchanged between different media (Fig. 2). The *ADH3* gene’s highest expression levels were obtained when cells were grown in the medium containing the non-fermentable carbon sources galactose (ratio of 0.141) and ethanol (ratio of 0.102) (Fig. 2). When glucose was used as a carbon source, the *ADH3* of *D. bruxellensis* was expressed but had the lowest value, which decreased 2.3 times and 3.0 times (in YNB + 2 % glucose and YNB + 10 % phosphate, respectively) in comparison to the medium with ethanol. As in *S. cerevisiae*, this gene in *D. bruxellensis* was found to carry recognition sites for the transcription factor Mig1 (Table S9, Supplementary Material) and is subjected to glucose repression. The expression of *ADH3* of *D. bruxellensis* was not fully repressed on glucose, similar to *S. cerevisiae ADH3* expression results (Young and Pilgrim 1985). Thus, *ADH3* of *D. bruxellensis* was suspected of influencing the ethanol production from glucose. The *DbGAL7* gene was also differentially expressed and showed its highest expression level when cells were grown in medium containing galactose (ratio of 0.159), which is similar to the data reported before for *D. bruxellensis* CBS 2499 strain (Moktaduzzaman et al. 2015). The effect of glucose repression in the growth medium on the expression of the *GAL7* gene was weaker than that on *ADH3*, as the *GAL7* expression on glucose was 1.4 times higher compared to *ADH3* (Fig. 2). The *DbPHO5* promoter did not manifest differences between the tested media, which indicates that this gene in the Y997 strain is not activated by phosphate depletion (Fig. 2).

The *D. bruxellensis* promoter *TEF1*, which showed the highest expression of all analysed promoters under all tested conditions, was further selected to build the expression vector.

### Overexpression of *D. bruxellensis ADH3* gene

We chose the *D. bruxellensis ADH*-encoding gene, gm1.2868_g (*ADH3,* Table S5, Supplementary Material) for the studies on its impact on ethanol production during aerobic and anaerobic glucose fermentation in *D. bruxellensis* as it was expressed albeit at low levels on glucose (Fig. 2). We were prompted to further investigate the role of *ADH3* in the fermentation of glucose by overexpressing this gene in *D. bruxellensis.* For this purpose, we cloned this gene, *D. bruxellensis ADH3*, under the strong and constitutive promoter *DbTEF1* (Fig. S4, Supplementary Material). The constructed plasmid P1227 (Fig. S4, Supplementary Material) was linearized by *Pst*I and used to transform the *ura3* mutant Y997. Among the obtained stable transformants (see Material and Methods and Stability assay of yeast transformants sections), the plasmid was shown to be present in one or two copies (Table S8, Supplementary Material). When we assume that the *ADH3* gene is present in the control strain in one copy, then the relative DNA copy number is between 1.25 and 2.15 (Table S8, Supplementary Material). These results suggest that the recipient strain is diploid and originally has two copies of *ADH3* in the genome. Then, the integration of one additional *ADH3* copy in the diploid genome will result in three copies and the relative ratio of 1.5 (*ADH3* transformants no.5, no.9 and no.11). The integration of two copies will result in four gene copies in the genome and in the relative copy ratio of 2 (*ADH3* transformants no.3 and no.10) (relative copy number, Table S8, Supplementary Material).

To verify the plasmid insertion site in the genome, the total DNA of one of the transformants (*ADH3* transformant Y997_no. 3) was digested with *Bgl*II (absent on the plasmid), self-ligated and transformed into bacteria to isolate the plasmid carrying genomic loci around the integration site (Fig. S6, Supplementary Material). As expected from the plasmid copy estimation (it is assumed that it carries two plasmid copies in the genome, Table S8, Supplementary Material), two kinds of plasmids were isolated carrying different genomic loci of *D. bruxellensis*. The sequencing of both plasmids confirmed that two copies of the integrated plasmid did not disrupt any genes. One of the plasmids carried the choline phosphate cytydyltransferase and the second plasmid carried a hypothetical protein with both genes found in scaffold 2 in *D. bruxellensis* CBS 2499 (http://genome.jgi.doe.gov/Dekbr2/Dekbr2.home.html) (Fig. S7, Supplementary Material).

**Fig. 3:**
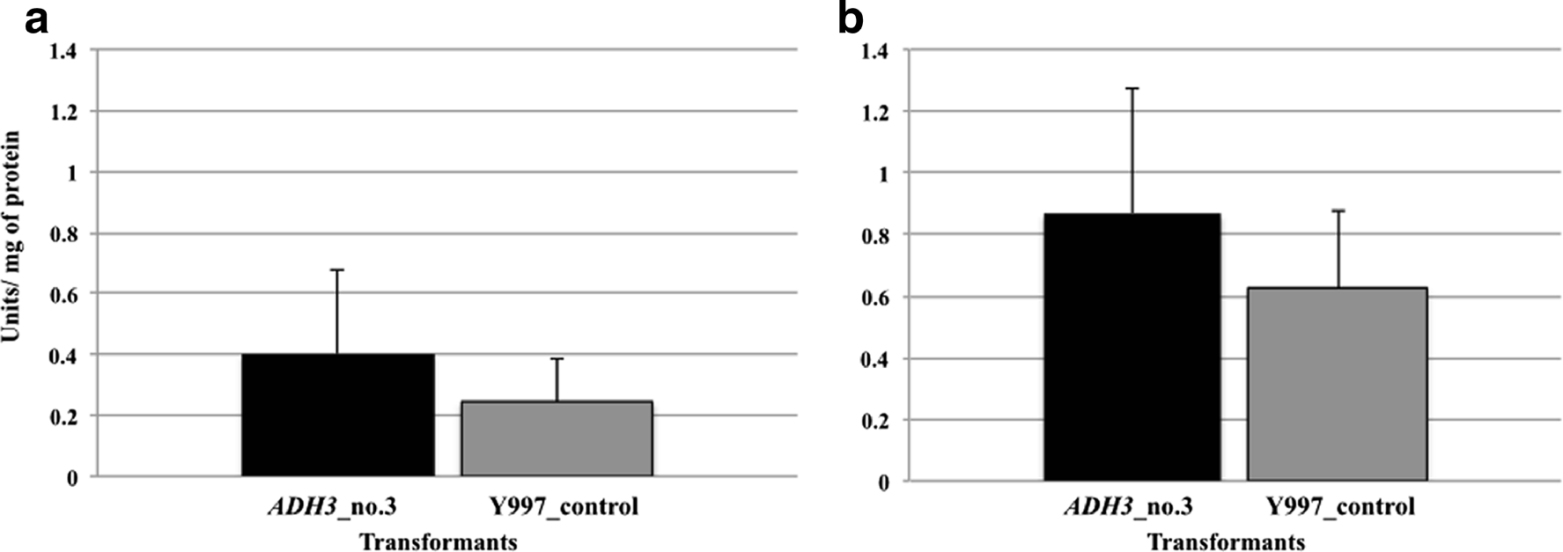
Specific activity of alcohol dehydrogenase in cell-free extracts measured in *D. bruxellensis* transformants. Ethanol (**a**) and acetaldehyde (**b**) were used as substrate. *ADH3*_no.3—*ADH3* transformant no.3, Y997_control—prototroph transformant carrying P892 (*empty vector*) integrated into the genome

The transformants carrying the *ADH3* expression plasmids displayed an elevated specific activity of alcoholdehydrogenase (Fig. 3), however, this increase was not statistically significant probably due to the activity of multiple isoforms of alcohol dehydrogenase.

**Fig. 4:**
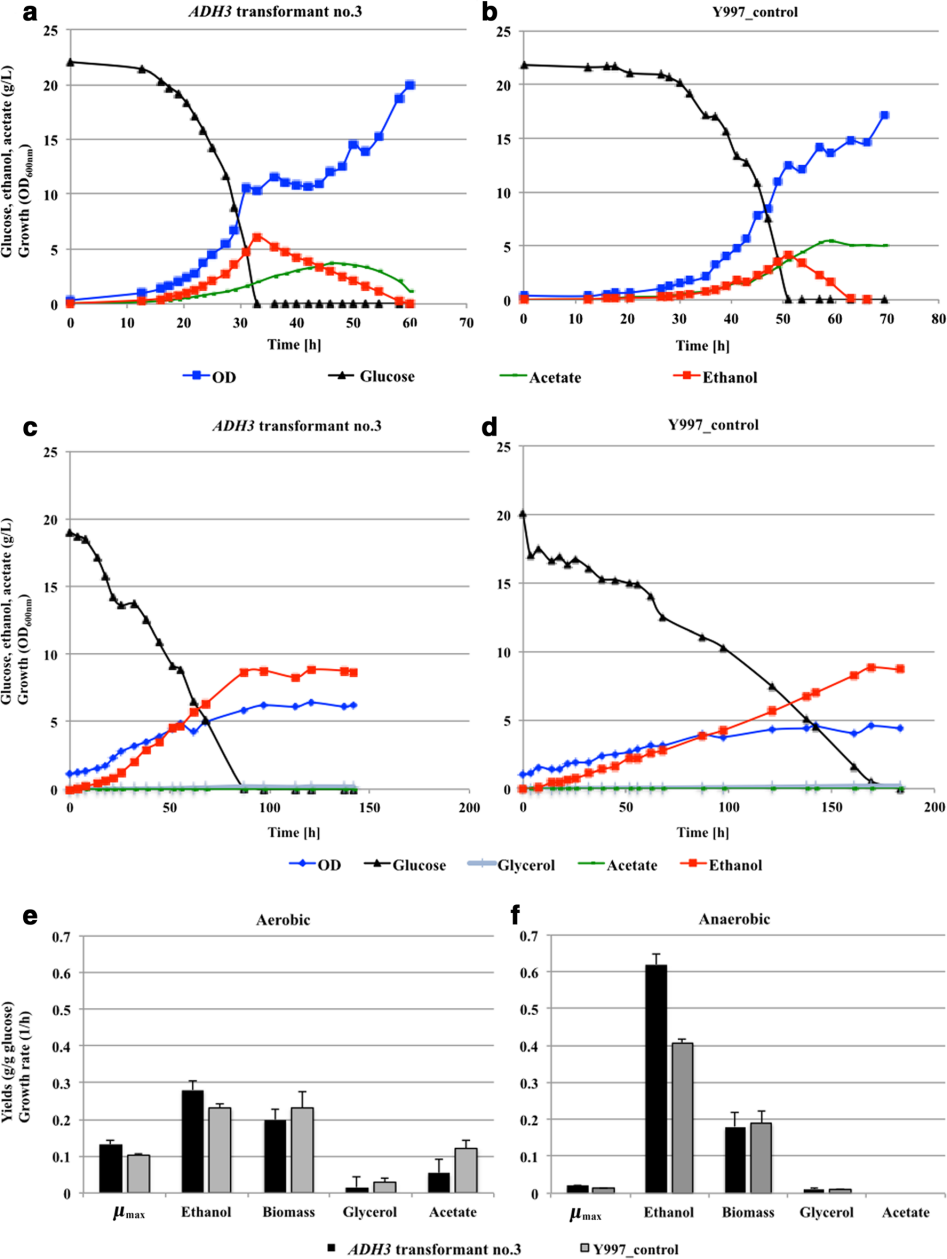
Glucose fermentation profiles of *D. bruxellensis* transformants in bioreactors in glucose-based minimal medium. Growth profiles of *ADH3* transformant no. 3 and Y997_control under aerobic (**a** and **b**) and anerobic (**c** and **d**) conditions (colour indications: *black* (glucose); *red* (ethanol); *green* (acetate), *blue* (OD_600nm_), glycerol (*grey*)). Yields of metabolites and maximum specific growth rate (μ_max_) under aerobic (**e**) and anaerobic (**f**) conditions (ethanol, acetate, glycerol and biomass) are shown in grammes per gramme of consumed glucose (g/g)

These transformants were grown under aerobic conditions in flasks or under controlled aerobic and anaerobic conditions in bioreactors and compared with Y997 strain carrying P892 integrated (empty vector, Y997_control). The results of the fermentation experiments in flasks showed that all stable transformants tested, carrying the *ADH3* expression plasmid, produced more ethanol than the control strain (data not shown). Since shake flasks conditions are not constant, the fluctuation in the concentration of dissolved oxygen is unpredictable over time (McDaniel et al. 1965; Sommerville and Proctor 2013), which may effect the alcoholic fermentation that depends on oxygen tension. We therefore studied one of the transformants (*ADH3* transformant no. 3) under fully controlled aerobic and anaerobic batch culture conditions in bioreactors. Under aerobic conditions, we observed that the *ADH3* transformant no. 3 had a 1.3 times higher specific growth rate than the control strain (Fig. 4. Table S10, Supplementary Material). In addition, the rate of depletion of glucose was 1.4 times faster in the same respective order, culminating in glucose being depleted in the medium much earlier (approximately after 33 h for *ADH3* transformant no. 3 (Fig. 4a) as compared to control (after 57 h, Fig. 4b)). The transformant carrying the *ADH3* expression plasmid produced 1.5 times more ethanol in g/L than the control strain. The ethanol yield of *ADH3* transformant no. 3 was 1.2 times higher than the control strain. There was no clear difference in yield of biomass between *ADH3* transformant no.3 and the control strain (Fig. 4a and Fig. 4b; Table S10, Supplementary Material). The *ADH3* overexpressing transformant also had a slightly lower yield of acetate, suggesting the redirection of the carbon flux towards ethanol, perhaps a more efficient conversion of acetaldehyde than in the control strain (Fig. 4a, b).

Under anaerobic conditions, we observed the inhibition of fermentation, the “Custer effect”, which had been reported to occur for other *D. bruxellensis* strains under the same conditions (Pereira et al. 2012), as the tested strains displayed lower specific glucose consumption rates in comparison with the aerobic conditions. It is noteworthy that no supplements such as amino acids were added to the medium. While there was no clear difference in biomass yield between the transformant overexpressing the *ADH3* gene and the control, the ethanol yield was 1.5 times higher in the transformant (Fig. 4f). The difference between the glucose consumption rates between strains suggests that in the transformant the overexpression of the *ADH3* gene helps to ease the “Custer effect”, as the transformant consumed glucose 1.7 times faster than the control (Table S10, Supplementary Material), and this difference is bigger when we compare the aerobic bioreactor data (Table S10, Supplementary Material). Under anaerobic conditions, the *ADH3* transformant no. 3 depleted the glucose after 87 h of fermentation (Fig. 4c), while the control strain did not finish the glucose earlier than 183 h (Fig. 4d).

## Discussion

In our study, we developed new molecular biology tools (an auxotrophic transformation system and an expression vector) that we used for the metabolic engineering of *D. bruxellensis*.

Although *D. bruxellensis* is a yeast species of interest for both industry and basic research, the molecular biology tools for the manipulation of this yeast are not well developed. The transformation system for *D. bruxellensis* developed by Miklenic et al. (2013) is based on a dominant selection marker and non-homologous integration of DNA, resulting in stable transformants. This system’s transformation efficiency is 20 transformants per microgram of DNA (Miklenic et al. 2013). In our study, we developed an alternative protocol for the development of a transformation system using auxotrophic markers. Although 30 *D. bruxellensis* strains were mutagenized in our study, only 10 gave rise to auxotrophic mutants, which had a frequency below 0.1 %. *D. bruxellensis* lacks a simple haploid organization of the genome (Hellborg and Piskur 2009; Piskur et al. 2012), and diploid or triploid genomes are common states among different isolates of *D. bruxellensis* (Curtin et al. 2012; Borneman et al. 2014). This polyploidy suggests why it was difficult to isolate auxotrophic mutants. Among a few stable *D. bruxellensis* 5-FOA-resistant colonies, we identified one *ura3* and two *ura5* mutants by sequencing of the gene and/or by mutation complementation. Using a plasmid carrying the *D. bruxellensis URA3* gene, we obtained the transformation efficiency, which is 3.4 times higher on average comparing to the transformation system using a dominant selective marker by Miklenic et al. (2013). The transformation of *D. bruxellensis ura5* mutants by the heterologous *S. cerevisiae URA5* gene resulted on average in 14 transformants per microgram of DNA, suggesting that the heterologous *URA5* gene is not efficiently expressed in *D. bruxellensis*. These results indicate that to achieve higher transformation efficiency by integrative plasmids, native *D. bruxellensis* genes are useful as selective markers.

To develop expression vectors that can be used for expression of the gene of interest (e.g., *D. bruxellensis ADH3* gene), we analysed the activity of *D. bruxellensis* promoters. Promoters, both constitutive and inducible, have been intensively studied and analysed in the yeast *S. cerevisiae* (Ciriacy 1975; Blumberg et al. 1988; Lohr et al. 1995; Walther and Schüller 2001; Peng and Hopper 2002; de Smidt et al. 2008); however in *D. bruxellensis*, this was not explored before, due to the unavailability of the genome sequence until recently. Ten *D. bruxellensis* complete genome sequences (Curtin et al. 2012; Piskur et al. 2012; Borneman et al. 2014; Crauwels et al. 2014; Valdes et al. 2014) have become available during the last few years, contributing to the development of molecular biology tools for *D. bruxellensis*. Recent research by Rozpedowska et al. (2011) generated data on the expression of rapid growth- and respiration-associated genes; Moktaduzzaman et al. (2015) reported on the expression of genes involved in hexose transport, galactose metabolism, respiration, TCA and glyoxylate cycles and gluconeogenesis. In our study, among the four *D. bruxellensis* promoters studied, we selected the promoter *TEF1* to build an integrative expression vector. The *TEF1* gene proved to have the highest expression level, which was not significantly changed between the different conditions tested. The expression of another studied gene, *ADH3*, which was not fully repressed by glucose, prompted us to investigate its impact on the glucose metabolism and the fermentation capacity of *D. bruxellensis*.

Alcohol dehydrogenase genes are known to be the key enzymes of alcoholic fermentation. *D. bruxellensis* is a yeast species that did not undergo the whole genome duplication event and has a very small number of gene families duplicated (Piskur et al. 2012). The *ADH1*, *2*, *3* and *5* group is among these duplicated gene families, which in *S. cerevisiae* is important for the reversible conversion of aldehydes to ethanol. It was also found to have a lineage-specific duplication in *D. bruxellensis* (Piskur et al. 2012). In *D. bruxellensis*, there are three genes of *ADH1-5* group, which were recently duplicated and show high sequence similarity between each other (gm1.961_g, gm.1.3583 and gm1.2868_g) and are more phylogenetically related to *S. cerevisiae ADH3* (Piskur et al. 2012). In *S. cerevisiae,* the *ADH1*, 3, 4 and *5* genes are responsible for the reduction of acetaldehyde to ethanol during glucose fermentation; whereas, *ADH2* catalyses the oxidation of ethanol to acetaldehyde (Piskur et al*.*^2012^). The alignment of the *D. bruxellensis ADH3* amino acid sequence with the protein sequences of *S. cerevisiae ADH1*, *ADH2* and *ADH3* using EMBOSS ClustalW2 highlights the presence of mitochondrial-targeting motifs characteristic for group I long-chain alcoholdehydrogenases (Box II and Box III) (Pilgrim and Young 1987; Reid and Fewson 1994) and a GroES-like domain (Murzin 1996; Taneja and Mande 1999) for both *S. cerevisiae* and *D. bruxellensis ADH3* genes. In addition, *D. bruxellensis* Adh3 was found to carry a sequence similar to *S. cerevisiae* N-terminal mitochondrial-targeting signal Box I with some amino acid substitutions, which could indicate a mitochondrial localization. In order to overexpress one of the three *S. cerevisiae ADH3* orthologs of *D. bruxellensis* (gm1.2868_g), a plasmid carrying *URA3* as a selective marker and the corresponding *ADH3* gene with promoter *TEF1* driving its expression were integrated into the genome of *ura3* host strain Y997. The created stable transformants had the *ADH3* gene constitutively expressed at high level and had glucose fermentation capacity substantially improved. Our results showed an increase of 1.2 to 1.5 times in the ethanol yield from glucose under both aerobic and anaerobic conditions when cells carry two additional copies of *ADH3* gene integrated into the genome. The overexpression of the *ADH3* gene enhanced the glucose consumption and ethanol production rates and resulted in a higher ethanol yield. On the other hand, the acetate yield was slightly lowered comparing to the control strain under aerobic conditions.

Ethanol and acetaldehyde freely diffuse across membranes. In yeast mitochondria, Adh3 was shown to be involved in (i) the conversion of acetaldehyde to ethanol with reoxidation of mitochondrial NADH (ethanol-acetaldehyde shuttle) which is important under anaerobic growth (Bakker et al. 2000; Lertwattanasakul et al. 2009), (ii) protection from the toxic effects of ethanol, and (iii) cofactors recycling by the conversion of ethanol to acetaldehyde (Saliola et al. 2006; Suwannarangsee et al. 2012). Our data show that the overexpression of *D. bruxellensis ADH3* enhances the conversion of acetaldehyde to ethanol (higher ethanol and lower acetate yields) under aerobic glucose fermentation. Reoxidation of NADH by this enzyme could also improve the glucose consumption rate by providing more NAD^+^ for glycolysis. It is interesting to note that the overexpression of the *ADH3* in *O. polymorpha* also strongly activated glucose alcoholic fermentation (Suwannarangsee et al. 2012). The stronger effect on ethanol yield and glucose consumption rates of *ADH3* overexpression was observed under anaerobic fermentation. The overexpression of *ADH3* gene under anaerobiosis improved the glucose consumption by 1.7 times and made the “Custer effect” (inhibition of alcoholic fermentation under anaerobiosis (Scheffers 1966)) less pronounced. Under oxygen-limited conditions, the level of enzymes involved in the central carbon metabolism, which generate NADH, is higher than that oxidizing NADH in *D. bruxellensis* (Tiukova et al. 2013). These findings suggest that the overexpression of *ADH3* of *D. bruxellensis* contributes to resolving the NADH imbalance during the conditions of the “Custer effect” (van Dijken and Scheffers 1986; Tiukova et al. 2013), as it improves the NADH oxidation through ethanol synthesis from acetaldehyde.

Our study presents a set of new molecular biology tools for *D. bruxellensis* that can be used to manipulate this yeast. The creation of strains carrying the alcohol dehydrogenase expression plasmid is the first success in the improvement of ethanol fermentation by this industrially important yeast.

## Electronic supplementary material

ESM 1 (PDF 2638 kb)
