# Supplementary material for: Alcohol dehydrogenase gene ADH3 activates glucose alcoholic fermentation in genetically engineered Dekkera bruxellensis yeast
Source: Appl Microbiol Biotechnol. 2016 Jan 8;100:3219–31. doi: 10.1007/s00253-015-7266-x (PMC4786601; doi:10.1007/s00253-015-7266-x)
Supplement: Supplementary file 1 — (PDF 2638 kb) [file 253_2015_7266_MOESM1_ESM.pdf]

Journal: Applied Microbiology and Biotechnology

Alcohol dehydrogenase gene *ADH3* activates glucose alcoholic fermentation in genetically engineered *Dekkera bruxellensis* yeast

Anna Judith Schifferdecker[1]*, Juozas Siurkas[2], Mikael Rørdam Andersen[2], Dorte Joerck-Ramberg[2], Zhihao Ling[1], Nerve Zhou[1], James E. Blevins[3], Andriy A. Sibirny[4,5], Jure Piškur[1], Olena P. Ishchuk[1]*

[1] *Department of Biology, Lund University, Sölvegatan 35, Lund SE-223 62, Sweden*
[2] *Department of Systems Biology, Technical University of Denmark, DK-2800 Lyngby, Denmark*
[3] *Consulting statistician, Pinnmöllevägen 48, SE-24755 Dalby, Sweden*
[4] *Institute of Cell Biology, NAS of Ukraine, Drahomanov Street 14/16, Lviv 79005, Ukraine*
[5] *Department of Biotechnology and Microbiology, University of Rzeszow, Zelwerowizca 4, Rzeszow 35-601, Poland*

*Correspondence to:
Anna Judith Schifferdecker, E-mail: Anna.Schifferdecker@biol.lu.se
Olena P. Ishchuk, E-mail: Olena.Ishchuk@biol.lu.se
Department of Biology, Lund University, Sölvegatan 35, Lund SE-223 62, Sweden, Tel: +4646 2221445, Fax: +4646 2224113

Table S1. *D. bruxellensis* strains used for selection of auxotrophic strains.

Laboratory designation	Original strain designation	Chromosome number *	Mutagenesis applied UV	Mutagenesis applied EMS	Mutants obtained UV	Mutants obtained EMS
Y858	CBS 72	5	+	-	0	-
Y859	CBS 73	7	+	-	0	-
Y860	CBS 74	6	+	+	0	0
Y861	CBS 75	6	+	+	0	0
Y864	CBS 78	6	+	+	0	0
Y865	CBS 96	7	+	+	0	0
Y866	CBS 97	3	+	-	0	-
Y867	CBS 98	7	+	-	*ura*	-
Y869	CBS 1940	8	+	-	*ura*	-
Y870	CBS 1941	ND	+	-	0	-
Y871	CBS 1942	7	+	-	*ura*	-
Y872	CBS 1943	5	+	-	0	-
Y878	CBS 2336	6	+	-	*thr*	-
Y879	CBS 2499	7	+	+	*met, ade*	*ade*
Y880	CBS 2547	6	+	+	0	0
Y881	CBS 2796	7	+	+	0	*ura*
Y882	CBS 2797	4	+	-	0	-
Y883	CBS 3025	8	+	-	0	-
Y888	CBS 4459	6	+	-	*met*	-
Y891	CBS 4602	4	+	-	0	-
Y895	CBS 6055	7	+	-	0	-
Y897	CBS 8027	5	+	+	*lys, met, ura*	*ura*
Y899	CBS 4480	5	+	-	0	-
Y990	CBS 4481	5	+	-	0	-
Y901	CBS 4482	6	+	-	0	-
Y902	CBS 4601	5	+	-	*met*	-
Y906	CBS 4914	6	+	-	0	-

Y908	CBS 5206	7	+	-	*his*	-
Y911	CBS 5512	7	+	-	0	-
Y912	CBS 5513	8	+	-	0	-

* - Chromosome number was estimated by PFGE.

ND - not determined.

Table S2. Oligonucleotides used in this study. Restriction sites are underlined.

**Name**	**Sequence 5´ → 3´**
DbTEF1-XbaI-sense	gcgcTCTAGAGATTAGTTTCATTTATAGGAATTCTCC
DbTEF1-ADH3-antisense	GAATGATGTTAAGCTTCTGAACATTTTGAATACTAATTAGACCAAATG
DbADH3-TEF1-sense	CATTTGGTCTAATTAGTATTCAAAATGTTCAGAAGCTTAACATCATTC
DbADH3-SalI-antisense	cgcGTCGACGAATGGAGAAGAAGAATGAAAC
DbADH3-RT-sense	GAGCAATGTCACAGTCTGTGGAGTACG
DbADH3-RT-antisense	TCCATCAAAGAGAAAACCTTTGGAAGATCGG
DbGAL7-RT-sense	GTTGTCCCTTATTGGGCATTGTGGCC
DbGAL7-RT-antisense	ATGGTGGATAGAAATGAGCGTGGAACCAG
DbPHO5-RT-sense	GCATTTGCACGTTTTTTCAGGACTTCGGAAC
DbPHO5-RT-antisense	TGCCGCTTTTAACTTCGCATCTCTTACTAAAG
DbTEF1-RT-sense	TGCCACACTGCTCACATTGCTTGTAAATTC
DbTEF1-RT-antisense	TATCAGTCTTCTCAACGGATCTGACGACAC
α -tubulin-sense	CACACAACGCTTGAAAACGCTGATTGCAC
α -tubulin-antisense	GGACAACACTGGAGCGTAGGAAACCAATG
OL7	GCGGTCGACGAGTTTGTCAGAAGTAAAGTTGTTG
OL8	GGCCTGCAGGATGAGTTCCAGGTTTCAATAAAGG
URA3-sequencing-sense	CTCTCTGCAAGGGTTGTAGC

Table S3. Plasmids used in this study.

Plasmid, laboratory designation	Description	Reference
P892	*D. bruxellensis* integrative plasmid carrying *URA3* of *D. bruxellensis*	This study
P1227	*D. bruxellensis* integrative plasmid carrying *URA3* of *D. bruxellensis* and *ADH3* ORF (plus 300bp downstream ORF) fused with *TEF1* promoter	This study
P1228	*S. cerevisiae URA5* integrated in *D. bruxellensis* Y997 strain	Dr. Boretsky Y. Institute of Cell Biology, NAS of Ukraine, Lviv, Ukraine

Table S4. Auxotrophic strains generated in this study and their transformants.

Laboratory designation	Strain of origin	Name	Description
Y997	Y881	*ura3*	*D. bruxellensis ura3* deficient strain
Y1009	Y871	*ura5*	*D. bruxellensis ura5* deficient strain
Y1010	Y871	*ura5*	*D. bruxellensis ura5* deficient strain
Y2885	Y997	control	*D. bruxellensis* Y997 carrying linearized plasmid P892 integrated
Y2887	Y997	transformant no.3	*D. bruxellensis* Y997 carrying P1227 linearized by *Pst*I and integrated into the genome, clone number 3
Y2888	Y997	transformant no.5	*D. bruxellensis* Y997 carrying P1227 linearized by *Pst*I and integrated into the genome, clone number 5
Y2889	Y997	transformant no.10	*D. bruxellensis* Y997 carrying P1227 linearized by *Pst*I and integrated into the genome, clone number 9
Y2890	Y997	transformant no.9	*D. bruxellensis* Y997 carrying P1227 linearized by *Pst*I and integrated into the genome, clone number 10
Y2891	Y997	transformant no.11	*D. bruxellensis* Y997 carrying P1227 linearized by *Pst*I and integrated into the genome, clone number 11

Table S5. Gene homologs coding for alcohol dehydrogenase in *D. bruxellensis* CBS 2499 genome (JGI genome portal v2.0).

BLAST hit definition	Gene model transcript
jgi/Dekbr2/4552/gm1.961_g	gm1.961_g
jgi/Dekbr2/7174/gm1.3583_g	gm1.3583_g
jgi/Dekbr2/6459/gm1.2868_g, *ADH3*	gm1.2868_g

Table S6. Analysis of variance (ANOVA) for gene expression response measured by Ct of RT-qPCR.

Gene	Source	Degrees of freedom	Adjusted sum of squares	Adjusted mean square	F	*p*-value*
ADH3	Media	3	64.571	21.524	16.89	0.000
	Clone	7	36.470	5.210		
	Error	51	64.981	1.274		
GAL7	Media	3	33.463	11.1542	10.42	0.000
	Clone	7	24.278	3.4683		
	Error	51	54.612	1.0708		
PHO5	Media	3	8.766	2.922	1.56	0.210
	Clone	7	43.772	6.253		
	Error	51	95.359	1.870		
TEF1	Media	3	4.7313	1.5771	1.87	0.147
	Clone	7	21.9579	3.1368		
	Error	51	43.0533	0.8442		
YML085C (α-tubulin)	Media	3	2.451	0.817	0.59	0.627
	Clone	7	29.316	4.188		
	Error	51	71.122	1.395		

* - Minitab's *p*-value “0.000” means that “$p \leq 0.0005$”.

Table S7. Gene expression study by RT-qPCR of *D. bruxellensis* genes *ADH3, GAL7, PHO5, TEF1* and *YML085C* of eight clones of prototroph transformant of Y997 strain run in duplicates.

Media	Tested gene	Ct 1	Ct 2	Ct, mean*	Ampl 1	Ampl 2	Ampl, mean*	Range of expression ratio	Expression ratio, mean	Standard deviation of expression ratios
1% Ethanol	*ADH3*	23.2	23.5	23.35	1.75	1.73	1.74	[0.069, 0.145]	0.102	0.037
		20.6	21.1	20.85	1.78	1.76	1.77			
		21.7	22.6	22.15	1.72	1.65	1.69			
		22.0	21.9	21.95	1.80	1.70	1.75			
		18.9	18.9	18.90	1.90	1.54	1.72			
		21.5	21.8	21.65	1.72	1.81	1.77			
		20.7	21.2	20.95	1.78	1.71	1.75			
		19.5	19.7	19.60	1.87	1.66	1.77			
	GAL7	23.4	23.0	23.20	1.75	1.73	1.74	[0.066, 0.093]	0.075	0.012
		21.2	21.4	21.30	1.73	1.75	1.74			
		21.5	22.1	21.80	1.75	1.76	1.76			
		21.9	22.6	22.25	1.74	1.74	1.74			
		19.6	20.0	19.80	1.74	1.83	1.79			
		22.0	22.2	22.10	1.74	1.86	1.80			
		21.5	22.0	21.75	1.75	1.84	1.80			
		20.6	20.7	20.65	1.75	1.76	1.76			
	PHO5	24.3	23.3	23.80	1.75	1.77	1.76	[0.044, 0.124]	0.088	0.035
		21.7	22.0	21.85	1.68	2.11	1.90			
		20.4	22.0	21.20	1.73	1.66	1.71			
		22.2	22.9	22.55	1.65	1.77	1.71			
		19.0	19.5	19.25	1.78	1.7	1.74			
		21.6	21.7	21.65	1.99	1.72	1.86			
		21.2	21.1	21.15	1.71	1.79	1.75			
		19.3	19.9	19.60	1.99	1.7	1.85			
	TEF1	18.2	18.1	18.15	1.79	1.81	1.80	[0.929, 1.212]	0.905	0.300
		17.1	16.8	16.95	2.10	1.84	1.97			
		17.7	17.7	17.70	1.70	1.87	1.79			
		18.2	19.2	18.70	1.89	1.92	1.91			
		16.2	15.7	15.95	1.92	1.53	1.73			
		16.7	17.3	17.00	1.52	1.83	1.68			
		17.4	18.2	17.80	1.66	1.75	1.71			
		15.4	15.1	15.25	1.89	1.81	1.85			
	YML085C (α-tubulin)	17.8	19.0	18.40	1.76	1.73	1.75	Control		
		16.3	16.6	16.45	1.69	1.75	1.72			
		16.1	16.2	16.15	1.7	1.52	1.61			
		17.4	18.1	17.75	1.67	1.77	1.72			
		16.1	16.7	16.40	1.64	1.85	1.75			
		17.3	17.2	17.25	1.83	1.72	1.78			
		16.6	18.1	17.35	1.74	1.76	1.75			
		15.5	15.8	15.65	1.78	1.73	1.76			
Phosphate depletion	*ADH3*	24.3	24.1	24.20	1.60	1.68	1.64	[0.025, 0.047]	0.035	0.010
		22.4	22.8	22.60	1.71	1.75	1.73			
		22.0	22.2	22.10	1.74	1.74	1.74			
		23.0	22.9	22.95	1.61	1.73	1.67			
		24.4	25.1	24.75	1.79	1.67	1.73			
		22.9	23.6	23.25	1.81	1.78	1.80			
		24.0	25.1	24.55	1.70	1.74	1.72			
		22.4	22.7	22.55	1.74	1.68	1.71			
	GAL7	23.4	23.5	23.45	1.66	1.76	1.71	[0.029, 0.076]	0.048	0.019
		21.0	21.7	21.35	1.73	1.79	1.76			
		21.1	21.9	21.50	1.76	1.72	1.74			
		23.1	23.2	23.15	1.79	1.75	1.77			

		23.2	23.8	23.50	1.76	1.72	1.74			
		22.5	22.8	22.65	1.68	1.76	1.72			
		24.6	25.1	24.85	1.77	1.69	1.73			
		21.3	21.9	21.60	1.68	1.79	1.74			
		23.4	23.2	23.30	1.79	1.98	1.89	[0.059-	0.086	0.033
		21.9	22.0	21.95	1.62	1.88	1.75	0.134]		
		21.7	22.3	22.00	1.73	1.64	1.69			
	PHO5	19.1	19.5	19.30	1.88	1.74	1.81			
		21.6	22.5	22.05	1.74	2.02	1.88			
		20.0	19.9	19.50	1.67	1.84	1.76			
		22.0	23.0	22.50	1.74	1.69	1.72			
		21.9	22.2	22.05	1.66	1.67	1.67			
		18.6	18.5	18.55	1.78	2.04	1.91	[0.510-	0.872	0.340
		16.3	15.9	16.10	1.89	1.64	1.77	1.303]		
		16.9	17.2	17.05	1.77	1.74	1.76			
	TEF1	17.1	17.1	17.10	1.96	1.82	1.89			
		17.6	18.2	17.90	1.54	1.71	1.63			
		17.7	17.8	17.75	1.76	1.80	1.78			
		18.6	19.7	19.15	1.75	2.34	2.05			
		15.7	16.0	15.85	1.68	1.74	1.71			
		18.8	19.9	19.35	1.75	1.81	1.78	Control		
		15.9	16.5	16.20	1.71	1.76	1.74			
		15.5	15.6	15.55	1.72	1.75	1.74			
	YML085C	16.1	16.4	16.25	1.66	1.73	1.70			
	(α-tubulin)	17.1	19.6	18.35	1.61	1.58	1.60			
		15.9	16.0	15.95	1.75	1.68	1.72			
		17.9	19.8	18.85	1.73	1.67	1.70			
		15.8	16.3	16.05	1.74	1.68	1.71			
		22.6	22.7	22.65	1.76	1.76	1.76	[0.040,	0.141	0.070
		18.5	18.6	18.55	1.70	1.75	1.73	0.205]		
		22.0	22.4	22.20	1.79	1.71	1.75			
**2%**	*ADH3*	19.3	19.8	19.55	1.79	1.85	1.82			
**Galactose**		20.7	20.8	20.75	1.69	1.96	1.83			
		20.6	21.7	21.15	1.89	1.76	1.83			
		20.9	20.4	20.65	1.87	1.88	1.88			
		20.5	20.6	20.55	1.77	1.78	1.78	[0.122,	0.159	0.040
		17.5	20.0	18.75	1.88	1.78	1.83	0.220]		
		21.1	20.8	20.95	1.79	1.73	1.76			
	GAL7	21.2	20.8	21.00	1.70	1.85	1.76			
		19.6	19.7	19.65	1.78	1.78	1.78			
		21.2	20.9	21.05	1.77	1.83	1.80			
		21.7	22.3	22.00	1.81	1.72	1.77			
		22.3	22.8	22.55	1.75	1.80	1.78	[0.037,	0.065	0.030
		20.8	21.7	21.25	1.72	1.74	1.73	0.108]		
		22.3	22.6	22.45	1.73	1.71	1.72			
	PHO5	21.1	21.1	21.10	1.75	1.95	1.85			
		20.2	20.2	20.20	1.68	1.96	1.82			
		23.6	24.0	23.80	2.03	1.64	1.84			
		24.1	23.5	23.80	1.91	1.86	1.89			
		18.0	18.2	18.10	1.67	1.81	1.74	[0.475,	0.740	0.190
		17.2	17.0	17.10	1.67	1.81	1.74	0.886]		
		18.0	18.9	18.45	1.72	1.70	1.71			
	TEF1	17.4	18.0	17.70	1.51	1.73	1.62			
		15.8	17.0	16.40	1.66	1.81	1.74			
		18.0	18.6	18.30	1.80	1.83	1.82			
		18.0	19.0	18.50	1.70	2.00	1.85			
		17.2	15.7	16.45	1.70	1.75	1.73	Control		
		16.2	17.6	16.90	1.68	1.75	1.72			
	YML085C	16.7	18.2	17.45	1.74	1.71	1.73			
	(α-tubulin)	16.3	17.7	17.00	1.69	1.77	1.73			
		16.5	16.8	16.65	1.80	1.69	1.75			

		17.1	17.5	17.30	1.71	1.77	1.74			
		17.9	20.2	19.05	1.66	1.75	1.71			
		23.9	24.0	23.95	1.73	1.74	1.74	[0.036,	0.045	0.007
		23.3	23.5	23.40	1.77	1.66	1.72	0.053]		
		20.1	20.4	20.25	1.85	1.77	1.81			
**2%**	*ADH3*	22.9	23.0	22.95	1.89	1.65	1.77			
**Glucose**		22.8	23.2	23.00	1.84	1.77	1.81			
		24.9	25.1	25.00	1.78	1.81	1.80			
		20.9	22.1	21.50	1.94	1.79	1.87			
		21.9	23.2	22.55	1.78	1.65	1.72			
		22.8	23.1	22.95	1.76	1.74	1.75	[0.053,	0.066	0.014
		23.0	23.4	23.20	1.72	1.79	1.76	0.087]		
		20.2	20.8	20.50	1.79	1.77	1.75			
	GAL7	20.8	21.4	21.10	1.79	1.71	1.75			
		22.5	22.9	22.70	1.79	1.74	1.77			
		22.9	24.4	23.65	1.73	1.78	1.76			
		22.2	22.9	22.55	1.69	1.74	1.72			
		22.0	19.8	20.90	1.77	1.73	1.75			
		23.2	23.6	23.40	1.81	1.76	1.79	[0.047,	0.086	0.035
		22.7	23.3	23.00	1.80	1.77	1.79	0.127]		
		19.2	19.7	19.45	1.83	1.77	1.80			
	PHO5	20.1	21.4	20.75	1.66	1.76	1.71			
		19.7	19.9	19.80	1.67	1.75	1.71			
		24.3	25.8	25.05	1.64	1.74	1.69			
		20.4	20.5	20.45	2.30	1.77	2.04			
		21.5	22.1	21.80	1.75	1.68	1.72			
		18.0	18.3	18.15	1.65	1.76	1.71	[0.833,	0.939	0.120
		18.3	18.0	18.15	1.82	1.85	1.84	1.086]		
		16.8	16.6	16.70	1.93	1.89	1.91			
	TEF1	15.8	17.1	16.45	1.68	2.04	1.86			
		16.7	17.4	17.05	1.84	1.76	1.80			
		18.4	19.0	18.70	1.70	1.79	1.75			
		17.5	17.2	17.35	2.06	1.65	1.86			
		15.0	16.3	15.65	1.72	2.05	1.89			
		17.9	18.6	18.25	1.69	1.76	1.73	Control		
		17.2	17.6	17.40	1.82	1.68	1.75			
	YML085C	16.1	16.6	16.35	1.69	1.69	1.69			
	(α-tubulin)	16.3	16.2	16.25	1.82	1.56	1.69			
		15.6	15.9	15.75	1.71	1.68	1.70			
		20.2	20.4	20.30	1.67	1.69	1.68			
		16.5	17.2	16.85	1.52	1.76	1.64			
		15.9	16.3	16.10	1.78	1.56	1.67			

* - Mean values of both Ct and Amplification factor (Ampl) of 8 clones were used to calculate the expression ratios by REST 2009 software v2.0.13 with RG mode (Pfaffl et al. 2002).

Table S8. DNA copy number of five transformants of Y997 carrying P1227 (*ADH3*_no. 3, 5, 9, 10, 11) and Y997 as control by RT-qPCR using *ADH3* primers (white) or α-tubulin primers (light grey).

Name	Ct values	Copy number[1], Ø mean value	Standard deviation (%)	Copy number normalized by tubulin	Relative copy number [2]
**transformant no.3**	10.7<br>11.1<br>10.3	165241406<br>125229568.3<br>218037342.4<br>Ø = 169502772.2	22	4	2
**transformant no.5**	11.3<br>10.8<br>11.3	109018671.2<br>154175683.4<br>109018671.2<br>Ø =124071008.6	17	2.5	1.25
**transformant no.9**	10.4<br>10.2<br>10.5	203436033.8<br>233686637.4<br>189812531.2<br>Ø =208978400.8	8.8	2.8	1.4
**transformant no.10**	10.1<br>10.9<br>10.5	250459136.5<br>143850999.1<br>189812531.2<br>Ø =194707555.6	22	4.3	2.15
**transformant no.11**	11<br>11.1<br>11	134217728<br>125229568.3<br>134217728<br>Ø =131221674.8	3.2	2.8	1.4
**control**	11.5<br>11.5<br>11.5	94906265.62<br>94906265.62<br>94906265.62<br>Ø =94906265.62	0	2	1
**transformant no.3_tubulin**	12.7<br>12.3<br>12.4	41310351.5<br>54509335.6<br>50859008.46<br>Ø =48892898.52	11.4	1	1
**transformant no.5_ tubulin**	12.6<br>12.4<br>12.6	44275338.47<br>50859008.46<br>44275338.47<br>Ø =46469895.13	6.7	1	1
**transformant no.9_ tubulin**	11.9<br>12<br>11.9	71925499.54<br>67108864<br>71925499.54<br>Ø =70319954.36	3.2	1	1
**transformant no.10_ tubulin**	12.2<br>12.3<br>12.3	58421659.36<br>54509335.6<br>54509335.6<br>Ø =55813443.52	3.3	1	1
**transformant no.11_ tubulin**	12.5<br>12.5<br>12.9	47453132.81<br>47453132.81<br>35962749.77<br>Ø =43623005.13	12.4	1	1
**control_ tubulin**	12.5<br>12.2<br>12.6	47453132.81<br>58421659.36<br>44275338.47<br>Ø =50050043.54	12	1	1

The samples were run in triplicates.

[1] DNA (gene) copy number was estimated by the following formula: $X = 2^{(38\text{-}Ct)}$; we assumed that Ct of 38 represents 1 copy of template in the sample to linearize the data.

[2] Relative DNA copy was estimated by dividing DNA copy number of *ADH3* strains to the control strain, which is assumed to carry one copy of tubulin gene (*YML085C*).

Table S9. Sequence analysis of *D. bruxellensis* promoters *ADH3, GAL7, PHO5, TEF1* (1000 bp upstream the open reading frame) for motifs Mig1, Cph1, Gal4, Adr1, Gcr1 and Cat8 using Python Script.

Promoter	Transcription factor	Transcription factor motif	Position in *D. bruxellensis* promoters (bp)	Consensus sequence of *S. cerevisiae*
DbADH3	Mig 1	CCGGGG	71	(G/C)(T/C)GGGG(G/A)G
DbGAL7	Mig 1	CTGGGG<br>CCGGGG	144<br>437	(G/C)(T/C)GGGG(G/A)G
DbPHO5	Pho4	CACGTG	835	CACGTG
DbTEF1	Gcr1	CTTCC	638<br>929	CTTCC

Table S10. Comparative physiology of the control strain (Y997_control) and the *ADH3* transformant no.3 under both strict aerobic and anaerobic conditions.

**Aerobic**			
	**Control**	**Transformant no.3**	**Heuristic *p*-values** [e]
μ $_{max}$ **(h^{-1})** [a]	0.103 ± 0.004	0.131 ± 0.014	0.008761222
**Yields (gg^{-1} glucose)** [b]			
Yse	0.232 ± 0.011	0.281 ± 0.025	0.014330907
Ysx	0.233 ± 0.045	0.199 ± 0.026	0.050616574
Ysac	0.122 ± 0.023	0.054 ± 0.034	0.182171028
Ysg	0.028 ± 0.014	0.015 ± 0.038	0.693786472
Ysp	0.001 ± 0.0001	0.001 ± 0.0001	0.501550232
**Specific rates (mM g^{-1}DWh^{-1})** [c,d]			
$q_{Ethanol}$	2.215 ± 0.336	3.993 ± 0584	0.002489248
$q_{Glucose}$	2.509 ± 0.222	3.608 ± 0.563	0.016359675
$q_{Acetate}$	0.696 ± 0.082	0.489 ± 0.168	0.073074346
$q_{Glycerol}$	0.006 ± 0.001	0.002 ± 0.001	0.227228771
**Anaerobic**			
μ $_{max}$ **(h^{-1})** [a]	0.014 ± 0.002	0.021 ± 0.002	0.00202181
**Yields (gg^{-1}glucose)** [b]			
Yse	0.408 ± 0.011	0.62 ± 0.029	8.2265E-05
Ysx	0.192 ± 0.031	0.18 ± 0.039	0.630375476
Ysac	0 ± 0	0 ± 0	1
Ysg	0.008 ± 0.001	0.011 ± 0.003	0.171346878
Ysp	0.003 ± 0.001	0.004 ± 0.001	0.137940841
**Specific rates (mM g^{-1}DWh^{-1})** [c,d]			
$q_{Ethanol}$	0.627 ± 0.036	1.623 ± 0.571	0.01298904
$q_{Glucose}$	0.393 ± 0.012	0.661 ± 0.198	0.029626465
$q_{Acetate}$	0 ± 0	0 ± 0	1
$q_{Glycerol}$	0 ± 0	0.01 ± 0.0002	0.209492224

Yields (ethanol, acetate, biomass, glycerol and pyruvate) were calculated during the exponential phase for as a function of glucose consumed as reported by van Hoek et al. (2000). Corresponding consumption and production rates were calculated during the same time intervals. Minimal media supplemented with 2% glucose was used in batch culture fermentations.

[a] Maximum specific growth rate.

[b] Yield coefficients per gram of glucose consumed (g^{-1}); Y_{se}, yield of ethanol; Y_{sx}, yield of biomass; Y_{sp}, yield of pyruvate; Y_{sac}, yield of acetate; Y_{sg}, yield of glycerol.

[c] Specific consumption rate per gram of biomass per hour (mmol g^{-1} h^{-1}); $q_{Glucose}$, glucose consumption rate.

[d] Specific production rates per hour per gram of biomass (mmol g^{-1} h^{-1});$q_{Ethanol}$, ethanol production rate; $q_{Acetate}$ and $q_{Glycerol}$ in the corresponding order.

[e] The *p*-values of t-tests are reported to quantify the evidence heuristically.

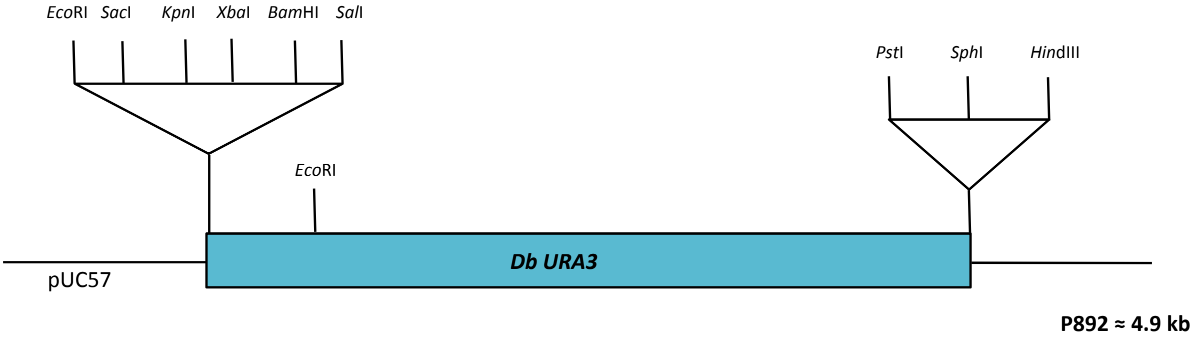

Fig. S1 The linear presentation of plasmid P892. The *URA3* gene of *D. bruxellensis* is shown as a blue box, the thin line represents pUC57. Restriction sites: RI, *Eco*RI; Sc, *Sac*I; K, *Kpn*I; Xb, *Xba*I; B, *Bam*HI; Sl, *Sal*I; P, *Pst*I; Sp, *Sph*I; H, *Hin*dIII.

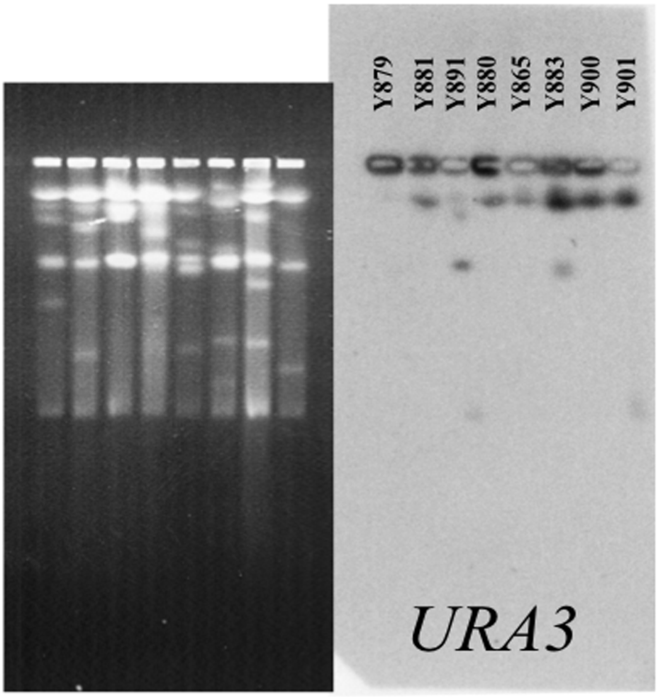

Fig. S2 Southern hybridization of *D. bruxellensis* strains using *URA3* gene as a probe. The PFGE gel is shown on the left, the hybridization membrane is shown on the right. Strains used: Y879, Y881, Y891, Y865, Y883, Y900 and Y901.

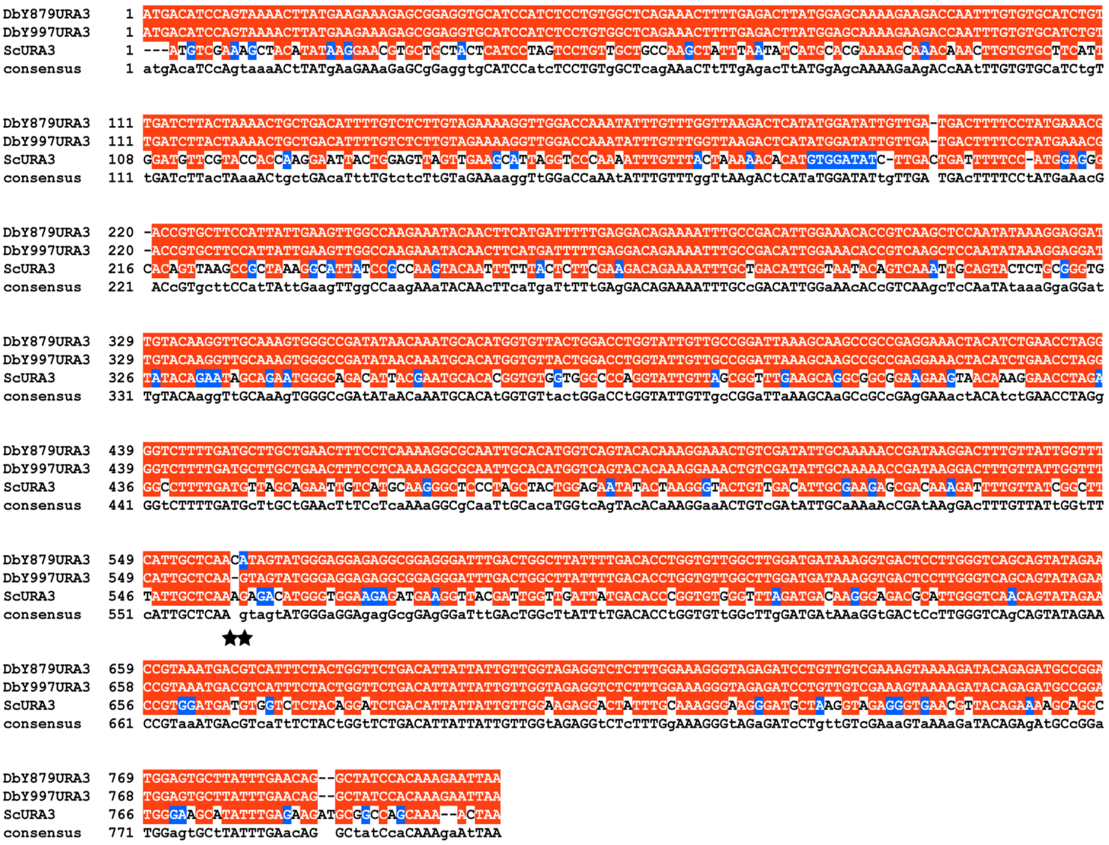

Fig. S3 Multiple alignment of the *URA3* gene nucleotide sequences of *D. bruxellensis* Y879 (DbY879), *D. bruxellensis* Y997 *ura3* (DbY997) and *S. cerevisiae* S288C (Sc) using ClustalW (http://www.ch.embnet.org/software/ClustalW.html) and BOXSHADE 3.21 (http://www.ch.embnet.org/software/BOX_form.html) software. Stars indicate the mutation site in *D. bruxellensis* Y997 *ura3* strain.

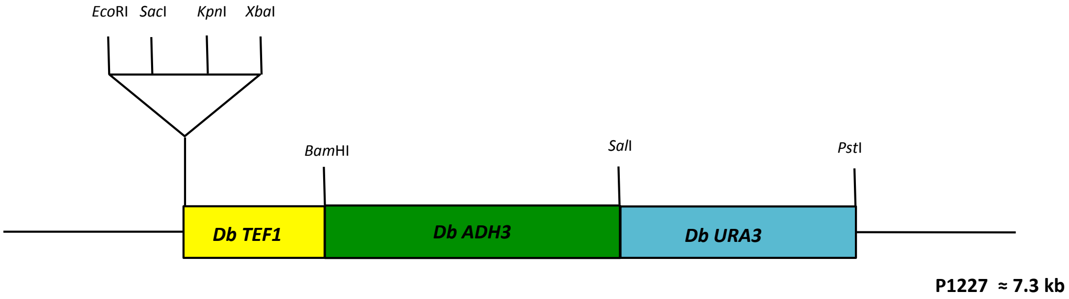

Fig. S4 The scheme of plasmids P1227 used for the overexpression of *D. bruxellensis ADH3* gene. Yellow box: *D. bruxellensis TEF1* promoter; green box: *D. bruxellensis ADH3* gene; blue box*: D. bruxellensis URA3* gene; thin line: pUC57. Restriction sites: *Bam*HI, *Eco*RI, *Kpn*I, *Pst*I, *Sac*I, *Sal*I, *Xba*I.

```
                          Box I
ADH1_S.c.       ---------------------------MSIPETQKGVIFYESHGKLEYKDIPVPKPKANE 33
ADH2_S.c.       ---------------------------MSIPETQKAIIFYESNGKLEHKDIPVPKPKPNE 33
ADH3_S.c.       MLRTSTLFTRRVQPSLFSRNILRLQSTAAIPKTQKGVIFYENKGKLHYKDIPVPEPKPNE 60
ADH3_Db         MFRSLTSFKT--QGPVLSRMALRLYS--TIPKTQKAMVFYKNGGPLKYEDIPVPKPKPSE 56
                                            :**:***.::**:. * *.::*****:**..*
                                                       Box II
ADH1_S.c.       LLINVKYSGVCHTDLHAWHGDWPLPVKLPLVGGHEGAGVVVGMGENVKGWKIGDYAGIKW 93
ADH2_S.c.       LLINVKYSGVCHTDLHAWHGDWPLPTKLPLVGGHEGAGVVVGMGENVKGWKIGDYAGIKW 93
ADH3_S.c.       ILINVKYSGVCHTDLHAWHGDWPLPVKLPLVGGHEGAGVVVKLGSNVKGWKVGDLAGIKW 120
ADH3_Db         ILINVRYSGVCHTDLHAWKGDWPLPTKLPLVGGHEGAGVVVACGSEVKNFKVGDYAGIKW 116
                :****:************:******.***************  *.:**.:*:** *****

ADH1_S.c.       LNGSCMACEYCELGNESNCPHADLSGYTHDGSFQQYATADAVQAAHIPQGTDLAQVAPIL 153
ADH2_S.c.       LNGSCMACEYCELGNESNCPHADLSGYTHDGSFQEYATADAVQAAHIPQGTDLAEVAPIL 153
ADH3_S.c.       LNGSCMTCEFCESGHESNCPDADLSGYTHDGSFQQFATADAIQAAKIQQGTDLAEVAPIL 180
ADH3_Db         LNGSCMGCEYCMQGAEPNCPKADLSGYTHDGSFQQYATADAVQAAHIPQGTDLAAAAPIL 176
                ****** **:*  * *.***.*************::*****:***:* ****** .****
                                        Box III
ADH1_S.c.       CAGITVYKALKSANLMAGHWVAISGAAGGLGSLAVQYAKAMGYRVLGIDGGEGKEELFRS 213
ADH2_S.c.       CAGITVYKALKSANLRAGHWAAISGAAGGLGSLAVQYAKAMGYRVLGIDGGPGKEELFTS 213
ADH3_S.c.       CAGVTVYKALKEADLKAGDWVAISGAAGGLGSLAVQYATAMGYRVLGIDAGEEKEKLFKK 240
ADH3_Db         CAGVTVYKALKTADLRPGQWVAISGAGGGLGSLAVQYAKAMGLRVVGIDGGSEKKELATK 236
                ***:******* *:* .*.*.*****.***********.*** **:***.*  *::*  .

ADH1_S.c.       IGGEVFIDFTKEKDIVGAVLKATDGGAHGVINVSVSEAAIEASTRYVRANGTTVLVGMPA 273
ADH2_S.c.       LGGEVFIDFTKEKDIVSAVVKATNGGAHGIINVSVSEAAIEASTRYCRANGTVVLVGLPA 273
ADH3_S.c.       LGGEVFIDFTKTKNMVSDIQEATKGGPHGVINVSVSEAAISLSTEYVRPCGTVVLVGLPA 300
ADH3_Db         LGAEEFIDFTQVSDVVKEMQNVTNGGPHGVINVSVSPRAMSQSVEYVRTLGKVVLVGLPA 296
                :*.* *****: .::*  : :.*.**.**:******  *:. *..* *. *..****:**

ADH1_S.c.       GAKCCSDVFNQVVKSISIVGSYVGNRADTREALDFFARGLVKSPIKVVGLSTLPEIYEKM 333
ADH2_S.c.       GAKCSSDVFNHVVKSISIVGSYVGNRADTREALDFFARGLVKSPIKVVGLSSLPEIYEKM 333
ADH3_S.c.       NAYVKSEVFSHVVKSINIKGSYVGNRADTREALDFFSRGLIKSPIKIVGLSELPKVYDLM 360
ADH3_Db         DAVVQTKVFDHVIKSIQIRGSYVGNREDTAEALDFFERGLVHSPIKVVGLSDLPKVFSLM 356
                .*   :.**.:*:***.* ******* ** ****** ***::****:**** **:::. *

ADH1_S.c.       EKGQIVGRYVVDTSK 348
ADH2_S.c.       EKGQIAGRYVVDTSK 348
ADH3_S.c.       EKGKILGRYVVDTSK 375
ADH3_Db         EKGKIAGRYVLDTSK 371
                ***:* ****:****
```

Fig S5 Alignment of the protein sequences of alcohol dehydrogenases genes of *S. cerevisiae* and *D. bruxellensis* (*ScADH1, ScADH2, ScADH3, DbADH3)* using EMBOSS Clystal W. Box I matches a N-terminal mitochondrial targeting signal, Box II a Zn-binding consensus sequence and Box III the NAD-binding domain pattern (Pilgrim et al. 1987).

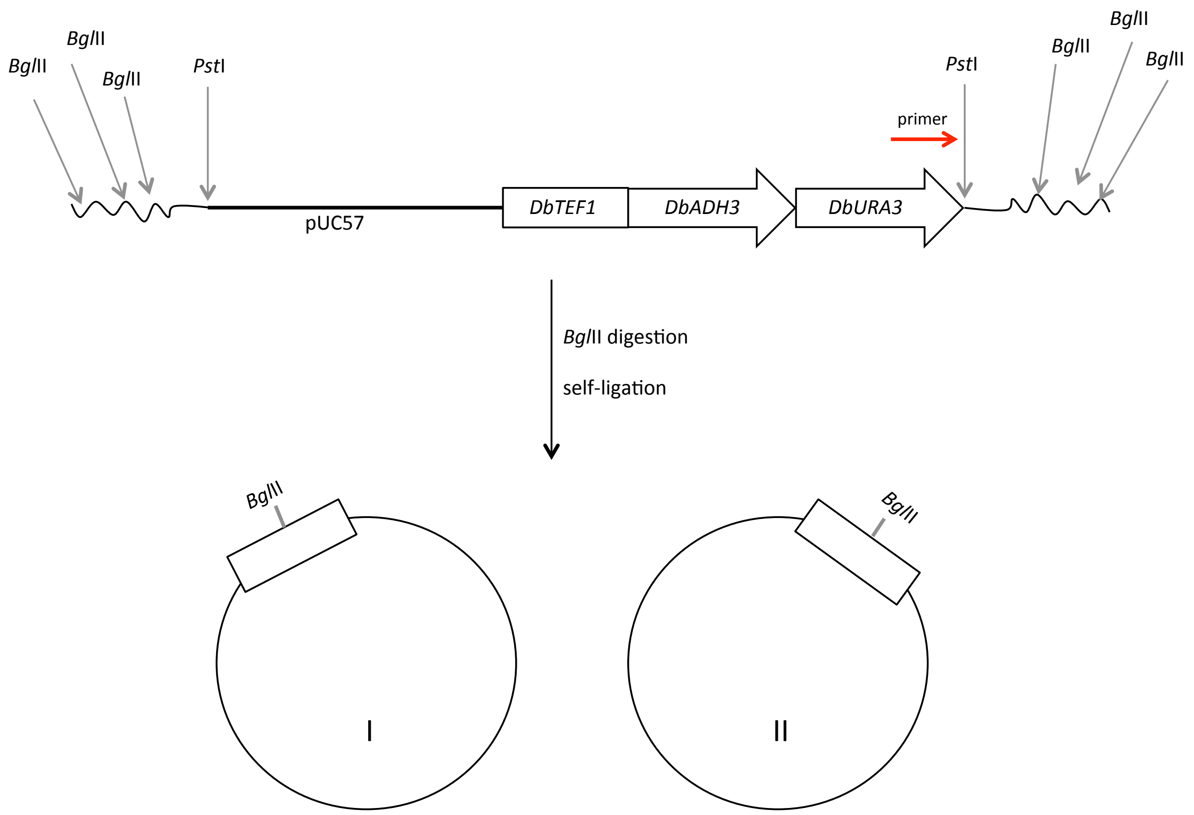

Fig. S6 Schematic illustration of the identification of the P1227 integration point in the *D. bruxellensis* genome. Total DNA of the transformant was digested with restriction enzyme *Bgl*II (which did not cut inside the P1227 plasmid), the digested DNA was self-ligated and transformed into TOP10 bacterial cells for the selection of ampicillin resistant clones. Subsequently, plasmids with *D. bruxellensis* genomic loci were isolated and sent for sequencing using *URA3*-sequencing-sense primer (red arrow). I and II represent two plasmids with different genomic loci.

Figure S7A

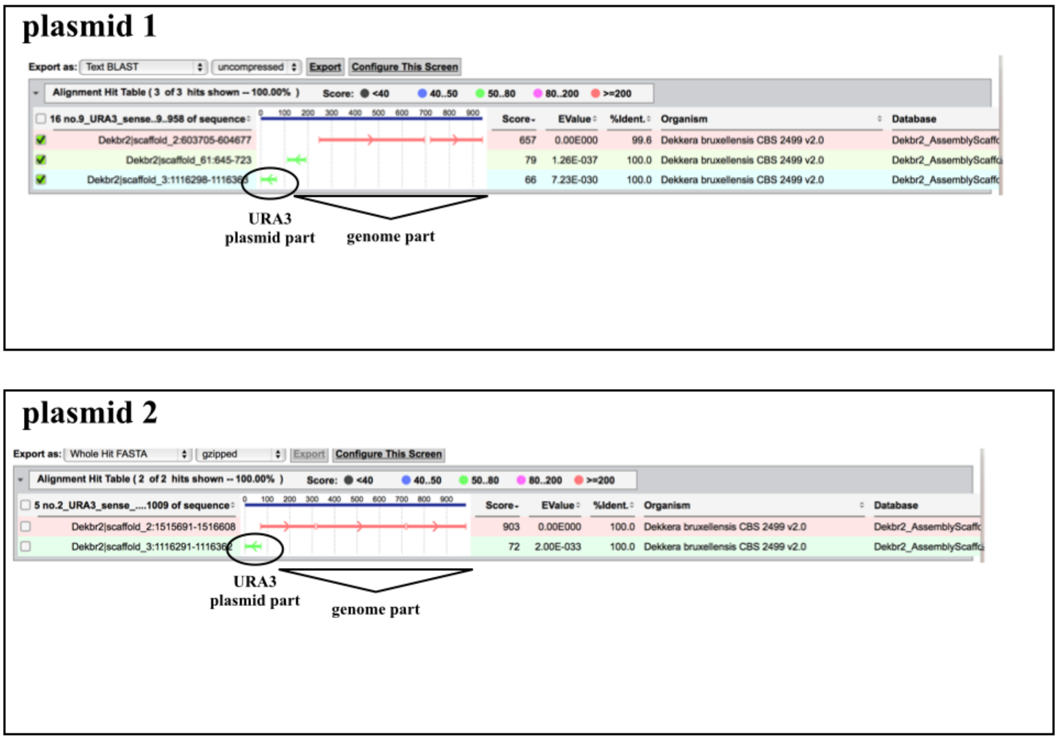

Figure S7B

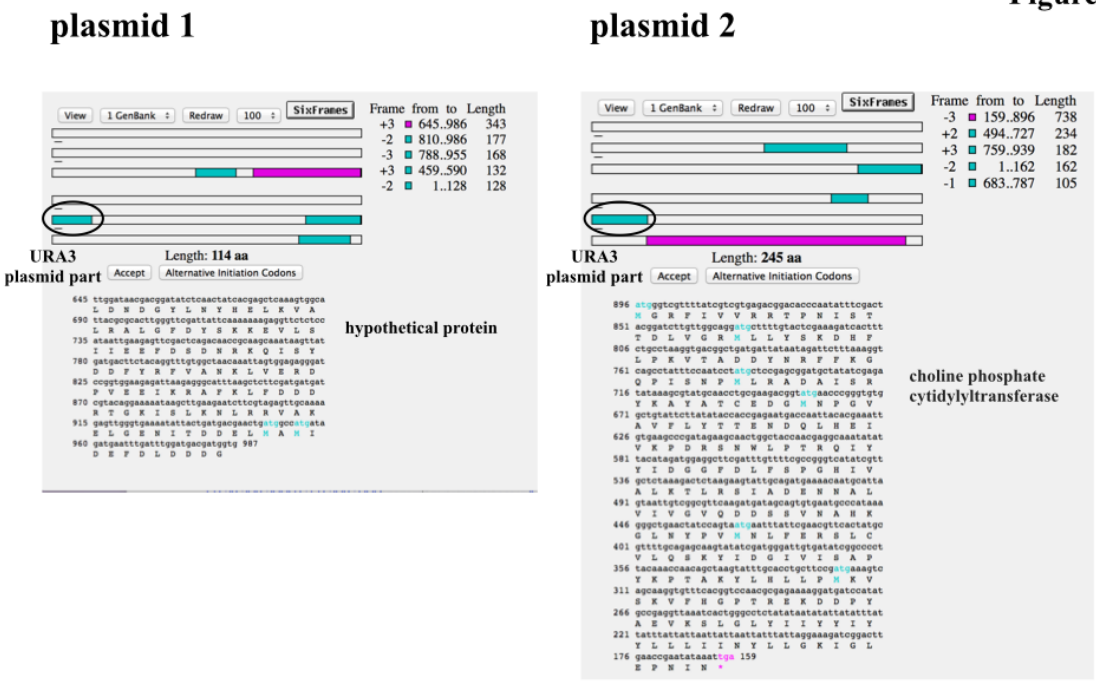

Fig. S7 Sequencing of the integration loci of P1227 in the genome. **A**: Alignment with genome sequence of *D. bruxellensis* CBS 2499. **B**: Search for putative genes using ORF finder (http://www.ncbi.nlm.nih.gov/gorf/orfig.cgi).

References

Pilgrim D, Young ET (1987) Primary structure requirements for correct sorting of the yeast mitochondrial protein *ADH* III to the yeast mitochondrial matrix space. Mol Cell Biol 7(1):294-304

Pfaffl MW, Horgan GW, Dempfle L (2002) Relative expression software tool (REST) for group-wise comparison and statistical analysis of relative expression results in real-time PCR. Nucleic Acids Res 30(9):e36

van Hoek P, van Dijken JP, Pronk JT (2000) Regulation of fermentative capacity and levels of glycolytic enzymes in chemostat cultures of *Saccharomyces cerevisiae.* Enzyme Microb Technol 26(9-10):724-736
